# *GABRA2* rs279858-linked variants are associated with disrupted structural connectome of reward circuits in heroin abusers

**DOI:** 10.1038/s41398-018-0180-0

**Published:** 2018-07-30

**Authors:** Yan Sun, Yang Zhang, Dai Zhang, Suhua Chang, Rixing Jing, Weihua Yue, Lin Lu, Dong Chen, Yankun Sun, Yong Fan, Jie Shi

**Affiliations:** 1 National Institute on Drug Dependence, Peking University, 100191 Beijing, China; 2 Department of Pharmacology School of Basic Medical Sciences, Peking University Health Science Center, 100191 Beijing, China; 3 National Laboratory of Pattern Recognition Institute of Automation, Chinese Academy of Sciences, 100190 Beijing, China; 4 Institute of Mental Health/Peking University Sixth Hospital and Key Laboratory of Mental Health, Peking University, 100191 Beijing, China; 5 Sanshui addiction treatment hospital, 528100 Guangdong, China; 6 Department of Radiology Perelman School of Medicine, University of Pennsylvania, Philadelphia, PA 19104 USA; 7 Beijing Key Laboratory on Drug Dependence Research, 100191 Beijing, China; 8 The State Key Laboratory of Natural and Biomimetic Drugs, 100191 Beijing, China; 9 The Key Laboratory for Neuroscience of the Ministry of Education and Health, Peking University, 100191 Beijing, China

## Abstract

The reward system plays a vital role in drug addiction. The purpose of this study is to investigate the structural connectivity characteristics and driving-control subnetwork patterns of reward circuits in heroin abusers and assess the genetic modulation on the reward network. We first defined the reward network based on systematic literature review, and built the reward network based on diffusion tensor imaging data of 78 heroin abusers (HAs) and 79 healthy controls (HCs) using structural connectomics. Then we assessed genetic factors that might modulate changes in the reward network by performing imaging-genetic screening for 22 addiction-related polymorphisms. The genetic association was validated by performing genetic associations (1032 HAs and 2863 HCs) and expanded-variant analysis. Finally, we estimated the association between these genetic variations, reward network, and clinical performance. We found that HAs had widespread deficiencies in the structural connectivity of the reward circuit (center in VTA-linked connections), which correlated with cognition deficiency. The disruptions synchronously were shown on the reward driving system and reward control system. *GABRA2* rs279858-linked variants might be a key genetic modulator for heroin vulnerability by affecting the connections of reward network and cognition. The role of the reward network connections that mediates the effects of rs279858 on cognition would be disrupted by heroin addiction. These findings provide new insights into the neurocircuitry and genetic mechanisms of addiction.

## Introduction

Heroin is one of the major addictive drugs that generates considerable public health concerns both in China and worldwide^1,2^. In recent decades, the prevalence of heroin abuse and on-medical prescription opioid use sharply increased in the USA and Europe^2,3^. The resurgence of heroin abuse has triggered dramatic increase in heroin overdose deaths^4^ and the spread of human immunodeficiency virus and hepatitis C virus infections^5^. Heroin misuse makes more attacks on the vulnerable population, such as adolescents and young adults, and can be life-threatening with significantly high morbidity and mortality. Adding new insight into the biological mechanism and identifying precise targets for neuromodulation are vital for the prevention and treatment of this devastating disease^6^.

Drug addiction including heroin addiction is seen as a disease of aberrant neuroadaptation in the brain reward system^7,8^. Despite the diversity in chemical structure and molecular targets, addictive drugs mediate their reinforcing properties by increasing dopamine (DA) concentrations in reward circuits, primarily in the ventral tegmental area (VTA) and its major projections (i.e., nucleus accumbens (NAc) and medial prefrontal cortex (mPFC))^8,9^. Drug addiction is hypothesized to develop from an imbalanced dual system in these reward circuits, the hyperactive reward motivation (mainly involves VTA and NAc) and hypoactive reward control (mainly involves the dorsolateral prefrontal cortex (dlPFC) and anterior cingulate cortex (ACC))^10,11^. This hypothesis has been validated in electrochemical and behavioral studies^12,13^. Recent advances in neuroimaging technology and brain network analysis allow us to investigate the reward circuit from a connectome perspective.

The reward processing in addicted individuals has been extensively studied by measuring brain reactivity to drug cues and non-drug rewards (mostly monetary). According to the systematic review we conducted to identify the disrupted reward circuit of substance abusers (details in Supplemental Method and Tables S1, S2), the most frequently reported regions mainly included the dlPFC, ACC, and subcortical areas (e.g., VTA and NAc), which we deemed as network nodes. We subsequently defined subnetworks for driving-control systems analysis based on authoritative reviews of addiction-related reward neurocircuitry^14,15^ (see Method section). Although the resting-state imaging studies have revealed that drug abusers exhibit functional connectivity deficiencies in the executive control system and impulsivity system^16,17^, it remains largely unknown how the reward driving-control system is affected by drug addiction.

Heroin primarily acts on endogenous opioid receptors and then triggers DA release in VTA and NAc, which are primarily modulated by GABA and the glutamate system^18^. Other neurotransmitter systems and neurotrophic factors (e.g., serotonin and brain-derived neurotrophic factor) may also be involved in this pharmacological processing^19^. Genetic factors contribute roughly 50% to the etiologies of addiction^20^. Since variants within the opioid-related genes may influence the various stages and endophenotypes of heroin addiction, it is important to understand how these genetic factors may modulate changes in the reward network of heroin addiction.

We evaluated heroin addiction-related genes and chose 22 variations as candidate genetic markers, which overlapped with SNPs reported in literature reviews associating genetic risk variants to opioid dependence^21,22^. These SNPs were in genes involved in the opioid system, DA system, GABA system, glutamate system, 5-hydroxytryptamine system, and other key polymorphisms. The supporting reasons for the selection of the 22 candidate SNPs are summarized in Table S3.

The present study aims to investigate the structural connectivity and driving-control subnetwork patterns of the reward circuit, and subsequently investigate the potential genetic modulation responsible for these changes. We also estimated the association between these genetic variations, reward network, and clinical performance.

## Methods and materials

### Study design

*Step 1*: We first investigated the reward network changes in heroin abusers (HAs). To better define the network nodes, we conducted systematic literature review and identified the most disrupted brain areas for rewarding stimuli in substance abusers. Then the reward network was built by probabilistic fiber tracking the diffusion tensor imaging (DTI) data from 78 HAs and 79 healthy controls (HCs). The changes in network characteristics and driving-control subnetworks were estimated.

*Step 2*: We then assessed genetic factors that may modulate changes in the reward network in HAs by performing imaging-genetic screening for 22 addiction-related polymorphisms. The genetic association was validated by performing genetic associations (1032 HAs and 2863 HCs) and expanded-variant analysis.

*Step 3*: Finally, the association between the genetic variation, changes in reward network, and clinical performance was analyzed by mediation analysis.

### Systematic review of imaging studies

The systematic literature review was conducted to help identify disrupted reward-related brain regions which responses to rewarding stimuli (substance-related stimuli, monetary rewards, and happy feelings) in substance abusers. The search terms included neuroimaging terms, substance addiction-related terms, and stimulus-related terms. The focus of the studies selected for the systematic review was on the comparisons between substance abusers and HCs and whole-brain analysis, which was published online before 30 November 2016. We identified a total of 65 studies and organized the findings by specific brain regions (Table S1). The search strategy is detailed in the Supplementary Materials.

### Subjects

#### MRI group

A cohort of 78 male HAs was recruited from drug addiction treatment centers and the local community in Zhongshan city, Guangdong province, China. They all met the criteria for heroin dependence based on the *Diagnostic and Statistical Manual of Mental Disorders*, 4th edition, but not for other substances (i.e., use of other opioids for not more than 1 month and other types of addictive drugs for not more than three times per year, with the exception of nicotine). Alcohol abusers were excluded from the study based on the Michigan Alcoholism Screening Test (score ≤ 4). In addition, we recruited 79 matched male HCs from the local community through newspaper advertisements. All of the participants were ethnic Han Chinese, and native to southern China. The HAs self-reported that they had no past or current major medical conditions and no personal or family history of major psychiatric disorders other than their current addiction. The exclusion criteria included (1) level of education < 9 years, (2) left-handed, and (3) contraindications for magnetic resonance imaging (MRI).

#### Genetic validation group

The genetic validation dataset included 1032 HAs (725 males, 307 females), who were recruited from multiple drug addiction treatment centers in Guangdong and Hubei provinces and 2863 HCs (1303 male, 1560 female) from the respective local communities. The MRI group was a subsample group derived from the genetic sample group (only from Zhongshan city). The characteristics of the subjects are summarized in Table 1.

**Table 1:** Demographics, addiction characteristics, and neurocognitive performance in study participants

Characteristic	Imaging and behavioral data			Genetic validation data		
	Heroin abusers (*n* = 78)	Healthy controls (*n* = 79)	*p-* value	Heroin abusers (*n* = 1032)	Healthy controls (*n* = 2863)	*p-* value
Age (years)	36.23 ± 3.94	37.52 ± 4.98	0.101	35.63 ± 6.61	31.69 ± 9.72	<0.001
Gender (male/female)	78/0	79/0	N.A.	725/307	1303/1560	<0.001
Cigarettes smoked per day	26.00 ± 9.09	8.72 ± 9.81	<0.001	24.11 ± 14.13	N.D.	N.D.
Heroin dosage (g/day)	0.52 ± 0.35	N.A.	N.A.	0.66 ± 0.61	N.A.	N.A.
Abstinence time (months)	5.39 ± 3.44	N.A.	N.A.	8.83 ± 6.32	N.A.	N.A.
Duration of heroin use (years)	15.10 ± 3.59	N.A.	N.A.	11.70 ± 6.32	N.A.	N.A.
Heroin craving at rest (score)	2.93 ± 0.24	N.A.	N.A.	N.D.	N.D.	N.D.
MoCA	21.92 ± 2.54	25.70 ± 2.88	<0.001			
IGT	−4.63 ± 21.07	4.02 ± 23.82	0.015			
BIS-11 (attention)	16.90 ± 2.72	15.91 ± 2.29	0.015			
BIS-11 (motor)	23.83 ± 4.47	21.08 ± 3.77	<0.001			
BIS-11 (non-planning)	27.40 ± 5.36	24.71 ± 5.02	0.001			
BIS-11 (sum)	68.13 ± 9.08	61.70 ± 8.75	<0.001			

*N.A*. not applicable, *N.D*. no data, *MoCA* the Montreal Cognitive Assessment, *BIS* the Barratt Impulsiveness Scale, *IGT* the Iowa Gambling Task

The data are expressed as mean ± standard deviation

The study was approved by the Peking University Institutional Review Board. All subjects were informed of the entire procedure and potential risks before being requested to sign a written informed consent form. All participants received monetary compensation for contributing to the study. None of HAs received systemic pharmacological substitution treatments during this study.

### Neurocognitive and behavioral assessments

MRI subjects underwent a detailed series of standardized neuropsychological tests, either on the same day or within 7 days of MRI scans. This included (1) the Montreal Cognitive Assessment (MoCA) to assess global cognitive ability, (2) the Barratt Impulsiveness Scale (BIS-11) to determine the level of impulsivity, (3) the Iowa Gambling Task (IGT) to evaluate decision-making ability, and (4) a visual analog scale to assess self-reported average heroin craving during the past week. Detailed descriptions of these assessments are available in the Supplementary Materials.

### MRI data acquisition and preprocessing

MRI was performed using a 1.5-T MR Signa HDxt imaging system (General Electric Medical System, Milwaukee, WI, USA) with a standard eight-channel head coil. Two experienced radiologists examined the T2 images, and no abnormalities were observed in the subjects. T1-weighted sagittal three-dimensional images were acquired with a spoiled gradient recalled echo sequence with coverage of the entire brain. For DTI, a total of 28 image sets was acquired with 56 axial slices (slice thickness: 2.4 mm with no gap; repetition time/echo time: 14.4 s/85 ms; 3 *b*0 images without diffusion weighting; 25 non-collinear diffusion-weighting gradients with *b* = 1000 s/mm^2^; acquisition matrix: 128 × 128; field of view: 256 × 256 mm^2^).

The DTI data were preprocessed using FSL5.0.7 (www.fmrib.ox.ac.uk/fsl/; accessed 6 September 2017) using the following steps: (1) Eddy-current and head motion correction, estimation of the diffusion tensors, and calculation of functional anisotropy (FA); (2) co-registration of T1 scans with their corresponding DTI *b*0 image so that ROIs that were defined in the structural MRI space could be transformed into native diffusion space; (3) the FA images were spatially normalized to the Montreal Neurological Institute (MNI) space using DARTEL of SPM8, resampled to 3 × 3 × 3 mm^3^ during the normalization, and smoothed with an 6-mm full-width at half-maximum Gaussian kernel.

### Network construction

The flow chart of network analysis was shown in Fig. S1.

#### Network nodes definition

**Fig. 1:**
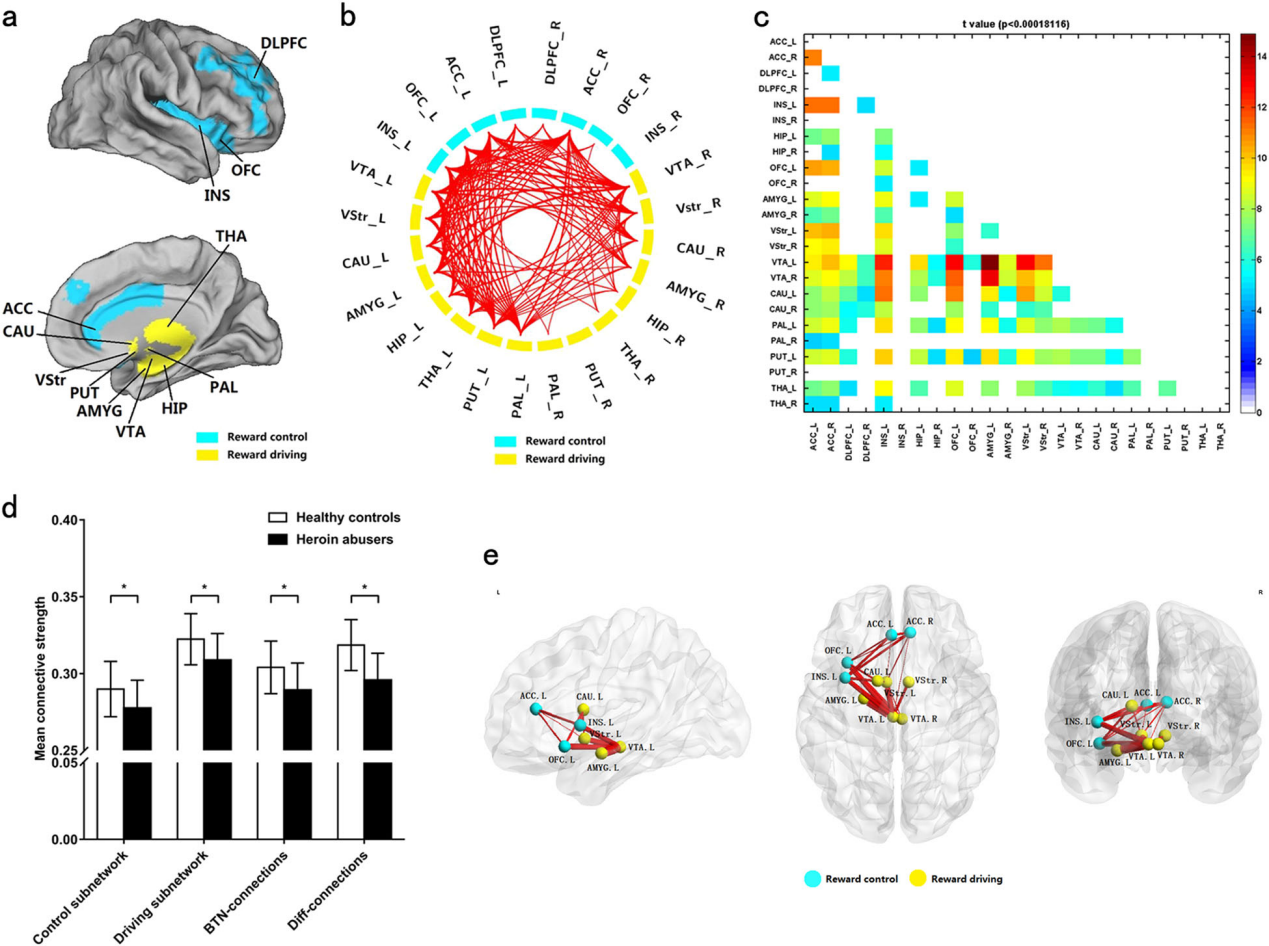
Significant deficiencies in the reward network of heroin abusers and the consequences to the subnetwork patterns in the reward network. **a** Two subnetworks of the reward network: reward control subnetwork (yellow) and reward driving subnetwork (blue). Abbreviations for each brain area are shown in Supplementary Table S2. **b** Among the 276 connections, 131 connections of the reward network had significantly lower connective strength in heroin abusers compared with healthy controls (Diff-connections, *p* < 0.05/276 after 10,000 permutation test). **c** *t-*values for all connections of the reward network. Details of connections with lower connective strength are summarized in Supplementary Table S4. **d** Compared with controls, heroin abusers presented significant decreases in mean connective strength in the reward control subnetwork, reward driving subnetwork, and connections between these two subnetworks (BTN-connections) and Diff-connections. The data are expressed as mean ± standard deviation. **e** The Diff-connections with *t*-value > 10 were located on VTA-linked connections

We defined the addiction reward-related brain regions (12 regions in each hemisphere), which were both highly reported in our systematic literature review and involved in biological mechanism of heroin addiction, as the nodes of reward network. These involved the dlPFC, ACC, VTA, orbitofrontal cortex (OFC), insula (INS), ventral striatum (VStr), caudate, amygdala (AMY), hippocampus (HIP), thalamus (THA), putamen, and pallidum (definitions are indicated in Table S2 and Fig. 1a). Specifically, we defined the subnetworks based on authoritative reviews^10,11,15^, the reward-driving subnetwork comprised the VTA, NAc, AMY, THA, and other subcortical regions that responded to impulsive motivation driving of substance reward (e.g., craving), and the reward-control subnetwork comprised the dlPFC, ACC together with OFC and INS that mainly exerted inhibitory control for substance reward.

#### Network edge definition

Between pairs of ROIs, structural connections were measured using the probabilistic fiber tracking method in FSL. This comprised of 276 structural connections for each subject (a symmetrical 24 × 24 matrix). Probabilistic fiber tracking was repeatedly sampled from the distributions of voxel-wise principal diffusion directions to generate a probabilistic streamline on the location of the true streamline and thereby build a connective value map in diffusion space. All the fibers were tracked with a curvature threshold of 0.2. Then, both the FA-value images and the probabilistic streamline images were warped from diffusion space into MNI space. The one-sample *t*-test was applied to the connective value maps of addiction subjects or HC subjects, respectively, to define streamline regions. The voxels were thresholded at *p* < 0.05 compared to zero. The connective strength of each edge was defined as the average FA values of all voxels that were included in the streamline regions.

#### Network analysis

The average connective strength of each edge was measured for each subject independently, including connections within the reward-driving subnetwork (Driving) and reward-control subnetwork (Control), connections between driving/control subnetworks (BTN-connections), and connections with significant differences between HAs and HCs (Diff-connections).

### DNA extraction and genetic variation detection

Genomic DNA was isolated from peripheral blood lymphocytes using the QIAamp DNA Mini Kit (Qiagen, Valencia, CA, USA). MassArray (Sequenom, San Diego, CA, USA) was used to genotype all markers using allele-specific Matrix-assisted laser desorption/ionization-time of flight mass spectrometry. Primers and multiplex reactions were designed using RealSNP.com website. None of the individual proportions significantly deviated from Hardy–Weinberg equilibrium (HWE) among HCs and HAs. The genotype call rate was >98% for each SNP.

### Statistical analysis

The statistical power of sample size computed by the Quanto soft (http://biostats.usc.edu/Quanto.html) was higher than 0.9. The variances were similar between the groups that were being statistically compared. Two-sample *t*-test was used to determine differences in demographic and clinical characteristics using SPSS 21.0 software. Each structural connection was compared between the HA and HC groups using 10,000 non-parametric permutation tests based on two-sample *t*-test scores, with “cigarettes smoked per day” and age as covariates. The threshold of Diff-connections was set at *p* < 0.00018116 (0.05/276) with multiple-comparison correction to identify statistically significant differences. The assessment of HWE was performed using the *χ*^2^ goodness-of-fit test. Additive linear regression was used for the association analysis of SNPs with structural connection, with “cigarettes smoked per day” and age as covariates using PLINK^23^. Linkage disequilibrium (LD) analyses were performed using Haploview 4.1 (http://www.broad.mit.edu/mpg/haploview/). The mediation analysis was performed using the model 4 in PROCESS^24^ to bootstrap the sampling distribution of the indirect effect.

## Results

### Demographic characteristics and neurocognitive performance

HAs significantly smoked more cigarettes per day and had lower MoCA and IGT scores, and higher BIS-11 scores compared with HCs. The age of HAs was significant older than controls in the genetic validation samples. Hence, the age and “cigarettes smoked per day” were included as covariates in the statistical analysis. The details of demographic and addictive characteristics (i.e., heroin dosage, duration of heroin use, and heroin craving) and neurocognitive performances are shown in Table 1.

### Characteristics of the reward network

Among the 276 total connections in the reward network, HAs presented a significant decrease in connective strength in 131 connections compared with HCs. These were mainly distributed in the left hemisphere (Diff-connections, *p* < 0.00018116 (0.05/276) with multiple-comparison correction; Fig. 1b, c, Supplementary Table S4). The significant decrease in connective strength of HAs was observed in the reward-driving subnetwork, reward-control subnetwork, Diff-connections, and BTN-connections of the reward network (Fig. 1d). No connections with higher strength were observed in HAs compared to HCs. Twenty Diff-connections with highest *t-*value were selected out (Fig. 1e). These connections mainly located on VTA-linked connections, specifically on VTA-NAC, VTA-AMY, and VTA-OFC.

### Genetic association analysis for the reward network

The screening of candidate genetic variants revealed only a significant effect for *GABRA2* rs279858 on the mean strength of Diff-connections of the reward network (*p*_main effect_ = 0.049, *p*_interactive effect_ = 0.012) (Table 2).

**Table 2:** Imaging genetic analysis of selected SNPs

Neurotransmitter and other related systems	Candidate loci	Variants	*p*-value			
			Genetic main effect	Gene by addiction interaction	Abusers	Controls
Opioid system	*OPRD1*	rs2234918	0.513	0.364	0.855	0.259
	*OPRK1*	rs1051660	0.758	0.241	0.294	0.551
	*OPRM1*	rs1799971	0.163	0.500	0.647	0.134
5-HT system	*HTR1B*	rs6296	0.942	0.522	0.597	0.683
		rs130058	0.965	0.781	0.880	0.812
	*5-HTT*	HTTPLR	0.551	0.672	0.480	0.900
Glutamate system	*GAD1*	rs3791878	0.544	0.460	0.349	0.930
	*GRIN2A*	rs1070487	0.740	0.814	0.695	0.960
		rs6497730	0.739	0.805	0.712	0.975
GABA system	*GABRA2*	rs279858	0.049*	0.012*	0.589	0.001*
	*GABRG2*	rs211014	0.456	0.811	0.507	0.701
DA system	*DRD2*	rs1079597	0.213	0.806	0.327	0.445
		rs1800497	0.206	0.592	0.222	0.558
	*DRD4*	VNTR	0.051	0.671	0.097	0.284
Other variants	*COMT*	rs4680	0.706	0.367	0.748	0.348
	*BDNF*	rs6265	0.978	0.354	0.553	0.479
	*NGFB*	rs2239622	0.745	0.285	0.324	0.633
	*CSNK1E*	rs135745	0.362	0.725	0.412	0.624
	*AVPR1R*	rs1587097	0.469	0.432	0.990	0.274
	*MAOA*	rs1137070	0.637	0.280	0.287	0.664
	*ZNF804A*	rs7597593	0.440	0.625	0.383	0.865
		rs1344706	0.987	0.857	0.905	0.891
*GABRA2 variants extended analysis*						
	Location	*GABRA2* variants	Genetic main effect	Gene by addiction interaction	Abusers	Controls
	Intron 9	rs693547	0.145	0.007*	0.357	0.003*
	Intron 8	rs519270	0.069	0.010*	0.494	0.002*
	Intron 7	rs279871	0.056	0.012*	0.589	0.001*
	Exon 5	rs279858	0.049*	0.012*	0.589	0.001*
*GABRA2*	Intron 4	rs279843	0.011*	0.022*	0.982	0.000*
	Intron 3	rs279827	0.042*	0.045*	0.885	0.003*
	Intron 3	rs10805145	0.027*	0.021*	0.804	0.001*
	Intron 3	rs9291283	0.105	0.196	0.674	0.038*
	Intron 1	rs11503014	0.159	0.972	0.354	0.296

^*^p < 0.05. The interactions were tested in a model which included main effects, with “cigarettes smoked per day” and age as covariates

**Fig. 2:**
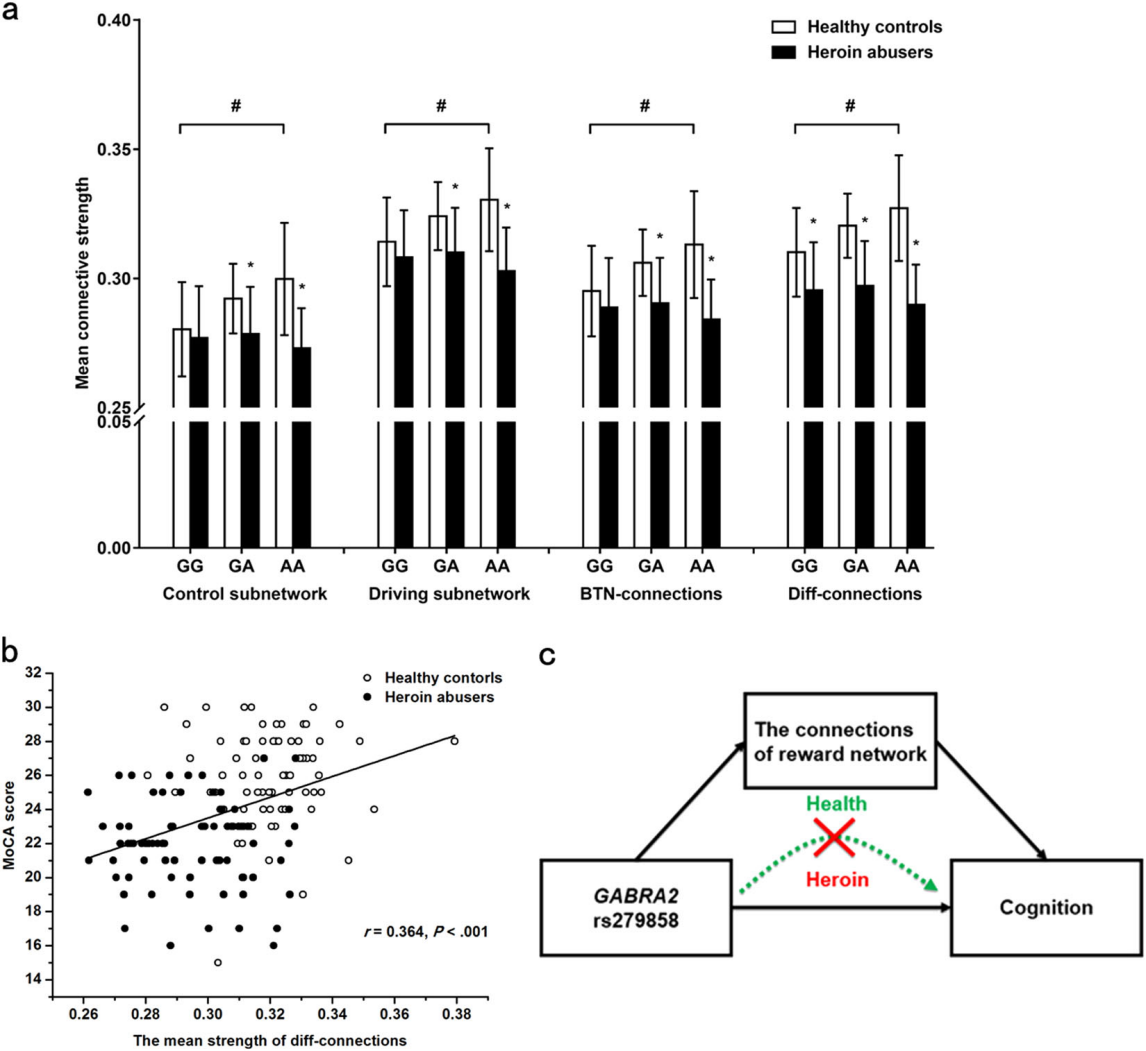
The association between *GABRA2* rs279858, reward network, and cognition. **a** The effect pattern of *GABRA2* rs279858 on the reward network. In the HC group, the mean connective strength in rs279858*G allele carriers was significantly lower than in A allele carriers. The A allele had a dose-dependent effect on the differences in connective strength of the reward network between HAs and HCs. *p < 0.05, within genetic group difference; ^#^*p* < 0.05, within health control group difference; BTN-connections, between reward control subnetwork and reward driving subnetwork. The data are expressed as mean ± standard deviation. **b** Significant positive correlation between the mean strength of Diff-connections and cognition, evaluated using MoCA. **c** The results of mediate analysis demonstrates the association between rs279858 and cognition was mediated by the connective strength of the reward network in the control group, but mediate association is indistinct after heroin use and addiction

The affect pattern of rs279858 on connections of reward network showed that the mean connective strength of rs279858*G allele carriers was significantly decreased compared to A allele carriers in the HC group. The rs279858*A allele had a dose-dependent effect on the differences in the connective strength of the reward network between HAs and HCs (Fig. 2a).

We further analyzed nine other SNPs in *GABRA2* that spanned intron 1 to intron 9. Two LD blocks were observed between these SNPs. The rs693547, rs519270, rs279871, rs279858, rs279843, rs279827, and rs10805145 SNPs, which were positioned in a separate LD block (D′ > 0.89, *r*^2^ > 0.71) with rs279858, had similar significant consequences on the mean connective strength of the reward network (Table 2, Supplementary Fig. 2).

Consequently, we validated the association between *GABRA2* rs279858 and heroin addiction in 1032 HAs and 2863 HCs. The genotype frequencies did not deviate from HWE (HA *p* is 0.92, HC *p* is 0.42). Significant differences in the allele and genotype frequencies of *GABRA2* rs279858 were observed between HAs and HCs. The rs279858*G allele frequency was significantly higher in HAs than HCs (*p* < 0.001, OR = 0.84, 95% confidence interval [CI] = 0.76–0.93; Table 3).

**Table 3:** Genetic distribution of *GABRA2* rs279858 in heroin abusers and health controls

Group	*N*	Genotype					Allele frequency				
		AA	AG	GG	*χ* ^2^	*p-* value	A	G	*χ* ^2^	*p-* value	OR (95% CI)
Heroin abusers	1032	202(0.20)	511(0.50)	319(0.31)	11.31	0.004*	915(0.44)	1149(0.56)	11.28	<0.001*	0.84(0.76–0.93)
Healthy controls	2863	688(0.24)	1409(0.49)	766(0.27)			2785(0.49)	2941(0.51)			

*OR* odds ratio, *CI* 95% confidence interval, *χ*^2^ test used for analysis of genotype frequency, *n* number of individuals

### The association between *GABRA2* rs279858, reward network, and cognition

The mean strength of Diff-connections was positively correlated with MoCA scores (*p* < 0.001, *r* = 0.364; Fig. 2b). No correlation was observed between the mean strength of Diff-connections and addiction characteristics, IGT scores, and BIS scores.

We then examined the role of the mean strength of reward network as a mediator for the association between *GABRA2* rs279858 with cognition. The indirect effect of rs279858 on cognition evaluated using MoCA through the mean strength of Diff-connections had a point estimate of −0.3289 and a 95% bias-corrected bootstrap confidence interval of −0.08673 to −0.0007 in HCs. This means the mediation effect was significant (similar findings observed for the reward-driving subnetwork and BTN-connections other than reward-control subnetwork as a mediator. Bootstrap estimates were based on 10,000 bootstrap samples). However, this association for rs279858, reward network, and cognition was not observed in HAs (Fig. 2c and Supplementary Table S5).

## Discussion

Our study in HAs detected widespread decrease in structural connectivity of the reward network, which were significantly associated with MoCA-assessed cognition levels. The HAs’ connections deficiencies of reward network mainly were observed in the VTA-linked connections, and in the reward-driving and reward-control subnetworks. We discovered that *GABRA2* rs279858-linked variants were associated with heroin addiction vulnerability as well as the connective strength of the reward network.

The advances in DTI tractography method allows us to delineate the neural axon fibers non-invasively based on the diffusion patterns of water molecules^25^. In our previous work using the whole-brain deterministic tracking analysis, we observed that HAs exhibited widespread increase in connective strength (fiber number-weighted) mainly in default-mode, attentional and visual systems^26^. However, the convergent changes in the reward circuit, the most critical system for addiction, were insufficient for analysis. The reward network has a significant amount of neural projections. Hence, we adopted the probabilistic fiber tracking method, which is more effective at determining the crossing fibers and intricate branching configurations than the deterministic approach^27^. By adopting the FA-weight probabilistic tracking approach, we observed the widespread decrease and no increase in connections in the reward network of HAs. These results were rather unexpected since it is generally considered that in addiction the reward-driving network was increased, whereas the reward-control network was decreased. Heroin use may trigger numerous forms of synaptic plasticity in the brain reward regions, and reduce inefficient and redundant neurons for acquiring sensitization toward addiction-related cues^28^. The neurotoxic effects of heroin are involved in loss of gray matter and white matter, cognition, neuronal apoptosis, synaptic defects, depression of neurogenesis, and so on^29–34^. The number of synapses, dendritic spines density, and membrane resistance of VTA dopaminergic neurons could be profoundly affected by chronic morphine use^35,36^. Consequently, HAs could have deficiency in myelination integrity and dendritic spine density of neural fibers within the reward network^37^, which reflected by the deficiency in DTI-based connectivities. The dynamic effects of heroin on reward network could be further investigated using HAs which had abused heroin for different time.

By systematic review of the literature for imaging studies, we found the highly reported brain areas of rewarding stimuli in substance addiction were mainly in the neurobiology of addiction^38^. We attempted to understand the neurobiological meaning of these changes through concerning the top and bilateral changes, which mainly presented at the VTA-centered core projections of the reward circuit. The VTA is considered as an essential center for reward and motivation^39^. The main reciprocal projections of the VTA are with NAc, mPFC (especially OFC), and AMY, and these connections form the core of the reward-loop^7^. Our finding suggests the main structural connections that are damaged in heroin addiction are presented at the core projections of the reward circuit, which parallel the reward driving system and reward control system. A possible explanation is that the initial drug-induced plasticity in the DAergic midbrain and subsequently in the VStr would recruit more dorsal striatal regions during chronic drug use and reshape the connectivity within these projections. This leads to compulsive drug dependence at the late stages of addiction^40^. Hence, the driving and control subnetworks of the reward circuit would have compact interactions and operate together to monitor reward processing.

GABA_A_ α2 receptor which encoded by *GABRA2* located on chr4p12^41^ was highly expressed in the mesolimbic DA reward pathway, including HIP and dopaminergic neurons in the substantia nigra and VTA^42^ (Supplementary Fig. 3a). *GABRA2* rs279858, a synonymous SNP in exon 5 of *GABRA2*, was the most common examined tag-SNP in the chr4p12 addiction-related region^43^. *GABRA2* rs279858 was associated with diversity of reward activation especially during adolescences^44^. The chromatin state data from the Roadmap Epigenomics Project^45^ demonstrated that there was a weak transcription and enhancer signal in the LD block of rs279858 in brain-related tissues (Supplementary Fig. 3b). The association between *GABRA2* rs279858*G and addiction risk has been reproducibly validated across different populations and different drug addictions (including heroin, alcohol, and cocaine)^46,47^. In line with previous studies, we provided evidence of the association between *GABRA2* rs279858 and variants in the rs279858-linked low-expression haplotype block with heroin addiction vulnerability^48,49^. GABA_A_ receptors may influence the opiates-reduced excitability of DA neurons within the reward pathway^50,51^ (GABA_A_ α2 receptors are highly expressed). The rs279858 variant may affect the distribution pattern of GABA_A_ α2 receptors^52^ that may be implicated in weaker neural connectivity in reward-loop and less inhibition of DA neurons, which results in the increased risk for addiction via developmental mechanisms^53^. However, addictive drug use could alter the expression of GABA_A_ subunits genes^54^. We have also speculated and assessed the epigenetic modifications of GABRA2 may be involved in the gene–heroin interaction. However, we have not found significant group difference for the epigenetic rate of CpG island in part of GABRA2 promoter, as well as its correlation with rs279858 and addiction characteristics. It was needed to detect more regions and pattern of epigenetic modulation of GABRA2 in the future. So, the overall interactive effects of heroin-by-rs279858 throughout the reward circuitry may be dynamic.

Cognitive impairment is a common risk associated with heroin addiction, which is aggravated by heroin use^55^. Here, we only observed general cognition abnormality in sober HAs associated with the strength of reward network, and not in decision making and impulsivity. A hypothesis to the deficiency of decision making and impulsivity in HAs would be reversible to some degree after abstinence, as we had demonstrated in our previous study^56^. However, damage of addiction on general cognition would be long-lasting and simultaneously occur with connectivity changes. Furthermore, *GABRA2* rs279858 accounted for significant changes to cognition, in part through affecting the mean strength of the reward network in healthy individuals. However, this association was indistinct in heroin users, which may be due to the detrimental effects of heroin after long-term use.

The present study does have some limitations. First, our imaging and behavioral data focused only on Han male subjects. However, according to the genetic data, there was much more men than women in the HA group, while much women than men in control group. This disequilibrium in gender distribution between HA and HC group is the limitation of this study. Second, we did not utilize whole-genome SNP analysis (e.g., GWAS) in our imaging-genetics screening. Hence, we could have missed critical SNP associations with heroin addiction. Third, the validation of the neurobiological mechanism underlying the consequences of *GABRA2* variants on the reward circuit could be performed using optogenetics and in vivo microscopy in preclinical models. Fourth, the study used a 1.5T MRI system, which was considered suboptimal by modern imaging standards. Other possible influence factors, such as the IQ and Socioeconomic Status, should be counter for in our future studies. Prospective and/or follow-up studies are needed to determine the causal role of *GABRA2* rs279858 in the reward circuit in heroin addiction.

## Conclusion

HAs have widespread deficiencies in the VTA-centered structural connectivity of the reward network, both in the reward-driving and reward-control systems. The *GABRA2* rs279858-linked SNPs were associated with susceptibility to heroin addiction by affecting the connections of the reward circuit and cognition. These findings provide new insights on how genetic variants influence the neurocircuitry that underlies individual heterogeneity to heroin addiction vulnerability. Our results provide evidence to garner interest in the scientific community to incorporate a circuit-level understanding of molecular and morphology changes that underlie drug responses.

## Electronic supplementary material

Supplemental material
