# Supplementary material for: GABRA2 rs279858-linked variants are associated with disrupted structural connectome of reward circuits in heroin abusers
Source: Transl Psychiatry. 2018 Jul 30;8:138. doi: 10.1038/s41398-018-0180-0 (PMC6066482; doi:10.1038/s41398-018-0180-0)
Supplement: Supplementary file 1 — Supplemental material [file 41398_2018_180_MOESM1_ESM.doc]

April 26, 2018, for *Translational Psychiatry*

**SUPPLEMENTARY MATERIAL**

***GABRA2* rs279858-linked variants** **are associated with disrupted** **structural connectome of the reward circuits in heroin abusers**

Yan Sun, PhD, MD^1^, *, Yang Zhang, MD^1, 2^ *, Dai Zhang, BS^3^, *, Suhua Chang, PhD^4^, Rixing Jin, BS^3^, Weihua Yue, MD^5^, Lin Lu, PhD, MD^1, 5^, Yankun Sun, PhD^1, 2^, Yong Fan, PhD^6, #^, Jie Shi, PhD, MD^1, 7, #^

*^1^**National Institute on Drug Dependence, Peking University, Beijing 100191, China*

^2^*Department of Pharmacology, School of Basic Medical Sciences, Peking University Health Science Center, Beijing 100191, China.*

*^3^**National Laboratory of Pattern Recognition, Institute of Automation,* *Chinese Academy of Sciences, Beijing 100190, China*

*^4^CAS Key Laboratory of Mental Health, Institute of Psychology, Chinese Academy of Sciences, Beijing 100101, China*

*^5^Institute of Mental Health/Peking University Sixth Hospital and Key Laboratory of Mental Health, Peking University, Beijing 100191, China*

*^6^Department of Radiology, Perelman School of Medicine,* *University of Pennsylvania, Philadelphia, PA 19104, USA*

*^7^Beijing Key Laboratory on Drug Dependence Research, Beijing 100191, China*

***Equally contributed to this work**

**^#^Correspondence:**

Prof. Jie Shi, National Institute on Drug Dependence, Peking University, 38 Xueyuan Road, Haidian District, Beijing 100191, China. Tel: 86-10-8280-1593, Fax: 86-10-6203-2624, Email: shijie@bjmu.edu.cn

or

Prof. Yong Fan, Department of Radiology, Perelman School of Medicine, University of Pennsylvania, Philadelphia, PA 19104, USA. Email: yong.fan@ieee.org

**This file includes:**

Supplementary Methods

Supplementary Table S1-5

Supplementary Figure S1-3

**Supplementary Methods**

*Neurocognitive assessments*

*Montreal Cognitive Assessment (MoCA).* The MoCA[^1^](#_ENREF_1) is a 30-point test that takes approximately 10-15 min to administer and less than 1 min to score. Items on this test include short-term memory recall (involving two learning trials of five nouns and delayed recall after approximately 5 min), visuospatial ability (assessed by a clock-drawing task and three-dimensional cube copying task), and multiple aspects of executive function (mainly assessed by an alternation task adapted from the trail-making B task). Attention, concentration, working memory, language, and orientation to time and place are also included in the scale. Higher MoCA scores indicate greater cognitive ability.

*Barratt Impulsiveness Scale (BIS-11).* The BIS-11[^2^](#_ENREF_2) is a 30-item self-report questionnaire that assesses impulsive personality traits in three dimensions: attention (inattention and cognitive instability), motor behavior (spontaneous actions), and non-planning (lack of forethought). Subjects were administered the Chinese version of the BIS-11. Higher BIS-11 scores indicate greater impulsiveness.

*Iowa Gambling Task (IGT).* We used the original card version of the IGT to evaluate decision-making performance. In this task, participants were presented with four different decks of cards and asked to select one card at a time, with the goal of maximizing their "jackpot" over 100 card choices. Unbeknownst to the participant, two of the four decks (C and D) are “advantageous”. Gains from these decks are relatively modest, but losses are modest as well, so choosing these decks consistently will result in an overall gain in the jackpot. The other two decks (A and B) are “disadvantageous.” Gains from these decks are high, but losses are even higher, so choosing these decks consistently will result in an overall loss from the jackpot. The outcome measure for this task is the net score (total number of selections from the advantageous decks minus total number of selections from the disadvantageous decks; [C+D] - [A+B]). Healthy individuals gradually switch their preference toward the advantageous decks and away from the disadvantageous decks. Subjects with better decision-making ability score higher.

***Visual Analog Scale (VAS).* We used** [**the VAS**](https://www.cebp.nl/vault_public/filesystem/?ID=1478) **to evaluate patients’ craving for heroin. The VAS consisted of one 10-point horizontal line anchored with “not at all” on one end and “extremely” on the other end. On these scales, subjects marked the part of the line that represented the extent of their average craving for heroin during the past week during the experimental period**[**^5^**](#_ENREF_5)**.**

*Systematic review of imaging studies*

We searched for studies in the literature that focused on neural responses to rewarding stimuli in substance addiction using PubMed, Web of Science, and Medline databases before November 30, 2016. The search terms included combinations of the following: (1) neuroimaging terms: MRI, magnetic imaging resonance, fMRI, functional MRI, BOLD, neuroimaging; (2) substance addiction-related terms: addiction, drug abuse, substance abuse, drug dependence, substance dependence, opiate, opioid, heroin, morphine, methadone, pethidine, cocaine, marijuana, cannabis, nicotine, tobacco, smoke, alcohol, methamphetamine, drug; and (3) stimulus-related terms: reward, cue, drug cue, craving, cue-reactivity, monetary reward, decision, decision-making, and pleasant. In addition, the bibliographies of all of the relevant papers were searched.

The studies meet the following inclusion criteria: (1) peer-reviewed original research articles in English language journals, (2) case-control studies in humans, (3) participants were diagnosed with substance addiction according to the *Diagnostic and Statistical Manual of Mental Disorders* criteria and healthy controls who had no history of psychiatric and neurological disorders, (4) studies that reported the results of whole-brain analyses in Talairach or Montreal Neurological Institute (MNI) space, and (5) samples with no overlap with the other included studies. In the case of studies that assessed the effects of a particular treatment, only the baseline condition was entered. Region-of-interest studies were also excluded. The targeted studies were extracted independently by two of the authors and checked mutually.

**Table S1. Reward-related brain areas associated with substance addiction.**

| **Brain region** | | **References** |
| --- | --- | --- |
| Cortical | Anterior cingulate cortex | [Li et al., 2015](#_ENREF_12)^6^; Li et al., 2012[^7^](#_ENREF_7); Li et al., 2013[^8^](#_ENREF_8); Artiges et al., 2009[^9^](#_ENREF_9); Nestor et al., 2011[^10^](#_ENREF_10); Tabatabaei-Jafari et al., 2014[^11^](#_ENREF_11); Canterberry et al., 2016[^12^](#_ENREF_12); Goudriaan et al., 2013[^13^](#_ENREF_13); Goldstein et al., 2009[^14^](#_ENREF_14); Goldstein et al., 2009[^15^](#_ENREF_15); Goldstein et al., 2010[^16^](#_ENREF_16); Potenza et al., 2012[^17^](#_ENREF_17); Luijten et al., 2012[^18^](#_ENREF_18); Luijten et al., 2011[^19^](#_ENREF_19); Zhang et al., 2011[^20^](#_ENREF_20); Fryer et al., 2013[^21^](#_ENREF_21); Tapert et al., 2004[^22^](#_ENREF_22); Wesley et al., 2011[^23^](#_ENREF_23); Filbey et al., 2016[^24^](#_ENREF_24); Gowin et al., 2014[^25^](#_ENREF_25); Monterosso et al., 2007[^26^](#_ENREF_26); Wrase et al., 2002[^27^](#_ENREF_27); Wexler et al., 2001 [^28^](#_ENREF_28) |
|  | Dorsolateral prefrontal cortex | [Li et al., 2015](#_ENREF_12)^6^; Li et al., 2012[^7^](#_ENREF_7); Yang et al., 2009[^29^](#_ENREF_29); Nestor et al., 2011[^10^](#_ENREF_10); Yalachkov et al., 2009[^30^](#_ENREF_30); Artiges et al., 2009[^9^](#_ENREF_9); Yip et al., 2016[^31^](#_ENREF_31); Wesley et al., 2014[^32^](#_ENREF_32); Luijten et al., 2012[^18^](#_ENREF_18); Zhang et al., 2011[^20^](#_ENREF_20); Lee et al., 2013[^33^](#_ENREF_33); Monterosso et al., 2007[^26^](#_ENREF_26); George et al., 2001[^34^](#_ENREF_34) ; Wrase et al., 2002[^27^](#_ENREF_27) |
|  | Insula | Gradin et al., 2014[^35^](#_ENREF_35); Jia et al., 2011[^36^](#_ENREF_36); Potenza et al., 2012[^17^](#_ENREF_17); Kobiella et al., 2014[^37^](#_ENREF_37); Luijten et al., 2011[^19^](#_ENREF_19); Zhang et al., 2011[^20^](#_ENREF_20); Nestor et al., 2011[^10^](#_ENREF_10); Yalachkov et al., 2009[^30^](#_ENREF_30); Artiges et al., 2009[^9^](#_ENREF_9); Ihssen et al., 2011[^38^](#_ENREF_38); Tapert et al., 2004[^22^](#_ENREF_22); Cousijn et al., 2013[^39^](#_ENREF_39); Nestor et al., 2010[^40^](#_ENREF_40); Gowin et al., 2014[^25^](#_ENREF_25); May et al., 2013[^41^](#_ENREF_41); Migliorini et al., 2013[^42^](#_ENREF_42); Stewart et al., 2015[^43^](#_ENREF_43) |
|  | Orbitofrontal cortex | [Li et al., 2015](#_ENREF_12)^6^; Li et al., 2012[^7^](#_ENREF_7); Yang et al., 2009[^29^](#_ENREF_29); Goudriaan et al., 2013[^13^](#_ENREF_13); Goldstein et al., 2009[^15^](#_ENREF_15); Tobler et al., 2016[^44^](#_ENREF_44); Young et al., 2014[^45^](#_ENREF_45); Ames et al., 2014[^46^](#_ENREF_46); Hermann et al., 2006[^47^](#_ENREF_47); Cousijn et al., 2013[^39^](#_ENREF_39); Filbey et al., 2016[^24^](#_ENREF_24); Wrase et al., 2002[^27^](#_ENREF_27); Wexler et al., 2001 [^28^](#_ENREF_28) |
|  | Inferior frontal gyrus | [Tabatabaei-Jafari et al., 2014](#_ENREF_21)^11^; [Yip et al., 2016](#_ENREF_27)^31^; [Konova et al., 2016](#_ENREF_10)^48^; [Rubinstein et al., 2011](#_ENREF_18)^49^; [Ames et al., 2013](#_ENREF_1)^50^; [Stewart et al., 2015](#_ENREF_20)^43^ ; Wexler et al., 2001[^28^](#_ENREF_28) |
|  | Precuneus | [Li et al., 2015](#_ENREF_12)^6^; [Li et al., 2013](#_ENREF_14)^8^; [Yang et al., 2009](#_ENREF_26)^29^; [Yip et al., 2016](#_ENREF_27)^31^; [Kobiella et al., 2014](#_ENREF_9)^37^; [Heinz et al., 2007](#_ENREF_8)^51^; [Wesley et al., 2011](#_ENREF_24)^23^ |
|  | Posterior cingulate cortex | [Li et al., 2013](#_ENREF_14)^8^; Nestor et al., 2011[^10^](#_ENREF_10); [Yip et al., 2016](#_ENREF_27)^31^; [Potenza et al., 2012](#_ENREF_17)^17^; [Konova et al., 2016](#_ENREF_10)^48^; [Yalachkov et al., 2013](#_ENREF_25)^52^; Park et al., 2007[^53^](#_ENREF_53) |
|  | Parahippocampus | [Li et al., 2015](#_ENREF_12)^6^; [Gradin et al., 2014](#_ENREF_7)^35^; [Li et al., 2012](#_ENREF_13)^7^; Artiges et al., 2009[^9^](#_ENREF_9); Yalachkov et al., 2009[^30^](#_ENREF_30); [Goldstein et al., 2009](#_ENREF_5)^15^; [Yalachkov et al., 2013](#_ENREF_25)^52^ |
|  | Precentral gyrus | [Konova et al., 2016](#_ENREF_10)^48^; Nestor et al., 2011[^10^](#_ENREF_10); [Stewart et al., 2015](#_ENREF_20)^43^; [Li et al., 2012](#_ENREF_13)^7^; [Goudriaan et al., 2013](#_ENREF_6)^13^; Canterberry et al., 2016[^12^](#_ENREF_12) |
|  | Fusiform gyrus | [Li et al., 2015](#_ENREF_12)^6^; [Yang et al., 2009](#_ENREF_26)^29^; [Yalachkov et al., 2013](#_ENREF_25)^52^; [Tapert et al., 2003](#_ENREF_22)^54^; Wrase et al., 2002[^27^](#_ENREF_27); Braus et al., 2001[^55^](#_ENREF_55) |
|  | Superior temporal gyrus | [Li et al., 2015](#_ENREF_12)^6^; [Luijten et al., 2011](#_ENREF_15)^19^; [Cousijn et al., 2013](#_ENREF_3)^39^; [Stewart et al., 2015](#_ENREF_20)^43^ ; Wexler et al., 2001[^28^](#_ENREF_28) |
|  | Superior frontal gyrus | [Yang et al., 2009](#_ENREF_26)^29^; [Stewart et al., 2015](#_ENREF_20)^43^; [Asensio et al., 2010](#_ENREF_2)^56^ ; Wexler et al., 2001[^28^](#_ENREF_28) |
|  | Superior parietal gyrus | [Li et al., 2015](#_ENREF_12)^6^; Yalachkov et al., 2009[^30^](#_ENREF_30); [Luijten et al., 2011](#_ENREF_15)^19^; [Wesley et al., 2011](#_ENREF_24)^23^ |
|  | Inferior parietal gyrus | [Li et al., 2015](#_ENREF_12)^6^; [Yalachkov et al., 2013](#_ENREF_25)^52^; [Fryer et al., 2013](#_ENREF_4)^21^; [Lee et al., 2013](#_ENREF_11)^33^ |
|  | Subcallosal gyrus | Yalachkov et al., 2009[^30^](#_ENREF_30); Artiges et al., 2009[^9^](#_ENREF_9); [Li et al., 2012](#_ENREF_13)^7^ |
|  | Middle temporal gyrus | [Konova et al., 2016](#_ENREF_10)^48^; [Tau et al., 2014](#_ENREF_23)^57^ |
|  | Inferior temporal gyrus | [Li et al., 2015](#_ENREF_12)^6^; [Yalachkov et al., 2013](#_ENREF_25)^52^ |
|  | Somatosensory cortex | [Yalachkov et al., 2013](#_ENREF_25)^52^ |
|  | Angular gyrus | [Goudriaan et al., 2013](#_ENREF_6)^13^ |
|  | Supplementary motor cortex | [Yalachkov et al., 2013](#_ENREF_25)^52^ |
|  | Lingual gyrus | [Tabatabaei-Jafari et al., 2014](#_ENREF_21)^11^ |
|  | Operculum | [Rubinstein et al., 2011](#_ENREF_18)^49^ |
| Subcortical | Ventral striatum | [Li et al., 2015](#_ENREF_12)^6^; Li et al., 2012[^7^](#_ENREF_7); Asensio et al., 2010[^56^](#_ENREF_56); Jia et al., 2011[^36^](#_ENREF_36); Tau et al., 2014[^57^](#_ENREF_57); Yalachkov et al., 2009[^30^](#_ENREF_30); Artiges et al., 2009[^9^](#_ENREF_9); Vaquero et al., 2016[^58^](#_ENREF_58); Bell et al., 2014[^59^](#_ENREF_59); Young et al., 2014[^45^](#_ENREF_45); Kobiella et al., 2014[^37^](#_ENREF_37); Luo et al., 2011[^60^](#_ENREF_60); Beck et al., 2009[^61^](#_ENREF_61); Beck et al., 2012[^62^](#_ENREF_62); Ihssen et al., 2011[^38^](#_ENREF_38); Vollstadt-Klein et al., 2010[^63^](#_ENREF_63); Wrase et al., 2007[^64^](#_ENREF_64); Nestor et al., 2010[^40^](#_ENREF_40); Malcolm et al., 2016[^65^](#_ENREF_65); van Hell et al., 2010[^66^](#_ENREF_66); Wrase et al., 2002[^27^](#_ENREF_27); van Hell et al., 2010[^66^](#_ENREF_66) |
|  | Thalamus | Li et al., 2012[^7^](#_ENREF_7); Li et al., 2013[^8^](#_ENREF_8); Zijlstra et al., 2009[^67^](#_ENREF_67); Asensio et al., 2010[^56^](#_ENREF_56); Beck et al., 2012[^62^](#_ENREF_62); Hermann et al., 2006[^47^](#_ENREF_47); May et al., 2013[^41^](#_ENREF_41); Wrase et al., 2002[^27^](#_ENREF_27); George et al., 2001[^34^](#_ENREF_34) |
|  | Hippocampus | Li et al., 2012[^7^](#_ENREF_7); Zijlstra et al., 2009[^67^](#_ENREF_67); Konova et al., 2016[^48^](#_ENREF_48); Artiges et al., 2009[^9^](#_ENREF_9); Kobiella et al., 2014[^37^](#_ENREF_37); Yalachkov et al., 2013[^52^](#_ENREF_52); Hermann et al., 2006[^47^](#_ENREF_47); Schneider et al., 2001[^68^](#_ENREF_68) |
|  | Caudate | [Li et al., 2015](#_ENREF_12)^6^; Gradin et al., 2014[^35^](#_ENREF_35); Li et al., 2012[^7^](#_ENREF_7); Li et al., 2013[^8^](#_ENREF_8); Jia et al., 2011[^36^](#_ENREF_36); Ames et al., 2013[^50^](#_ENREF_50); van Hell et al., 2010[^66^](#_ENREF_66); Rose et al., 2013[^69^](#_ENREF_69) |
|  | Amygdala | Li et al., 2012[^7^](#_ENREF_7); Yang et al., 2009[^29^](#_ENREF_29); Young et al., 2014[^45^](#_ENREF_45); Kobiella et al., 2014[^37^](#_ENREF_37); Fryer et al., 2013[^21^](#_ENREF_21); Schneider et al., 2001[^68^](#_ENREF_68); Filbey et al., 2013[^70^](#_ENREF_70) |
|  | Putamen | [Li et al., 2015](#_ENREF_12)^6^; Li et al., 2012[^7^](#_ENREF_7); Li et al., 2013[^8^](#_ENREF_8); Luijten et al., 2011[^19^](#_ENREF_19); Artiges et al., 2009[^9^](#_ENREF_9); Ames et al., 2013[^50^](#_ENREF_50); Braus et al., 2001[^55^](#_ENREF_55) |
|  | Ventral tegmental area | Yang et al., 2009[^29^](#_ENREF_29); Zijlstra et al., 2009[^67^](#_ENREF_67); Beck et al., 2012[^62^](#_ENREF_62); Filbey et al., 2016[^24^](#_ENREF_24) |
|  | Pallidum | [Li et al., 2015](#_ENREF_12)^6^ |

**Table S2.** **Abbreviations of the 12 brain areas in the reward network.**

| **Brain region** | **Abbreviation** | **Atlas** |
| --- | --- | --- |
| Anterior cingulate cortex | ACC | Harvard-Oxford Atlas |
| Dorsolateral prefrontal cortex | dlPFC | Brodmann's area |
| Insula | INS | Harvard-Oxford Atlas |
| Hippocampus | HIP | Harvard-Oxford Atlas |
| Orbitofrontal cortex | OFC | Harvard-Oxford Atlas |
| Amygdala | AMYG | Harvard-Oxford Atlas |
| Ventral striatum | VStr | Harvard-Oxford Atlas |
| Ventral tegmental area | VTA | peak coordinate (0, -16, -8) with radius of 2.5 mm[^71^](#_ENREF_71) |
| Caudate | CAU | Harvard-Oxford Atlas |
| Pallidum | PAL | Harvard-Oxford Atlas |
| Putamen | PUT | Harvard-Oxford Atlas |
| Thalamus | THA | Harvard-Oxford Atlas |

Table S3. Candidate genetic loci selected for reward network association in heroin addiction

| **Neurotransmitter and other related systems** | **Candidate gene** | **Variants** | **Effects on opioid or other substance addiction** | **References** |
| --- | --- | --- | --- | --- |
| Opioid system | *OPRD1* | rs2234918 | Associated with susceptibility to heroin addiction. | [^72^](#_ENREF_72) |
|  | *OPRK1* | rs1051660 | Associated with susceptibility to heroin addiction. | [^73-75^](#_ENREF_73) |
|  | *OPRM1* | rs1799971 | Associated with susceptibility to heroin addiction. | [^76-81^](#_ENREF_76) |
| 5-HT system | *HTR1B* | rs6296 | Associated with susceptibility to heroin addiction and amount of self-injected heroin. | [^82^](#_ENREF_82) |
|  |  | rs130058 | Associated with heroin addiction, verified by meta-analysis. | [^83^](#_ENREF_83) |
|  | *5-HTT* | HTTPLR | “S” promoter polymorphism associated with higher risk of opiate addiction, particularly in subjects with more consistent aggressiveness and impulsiveness. | [^84-86^](#_ENREF_84) |
| Glutamate system | *GAD1* | rs3791878 | Associated with susceptibility to heroin addiction. | [^87^](#_ENREF_87) |
|  | *GRIN2A* | rs1070487 | Associated with susceptibility to heroin addiction. |  |
|  |  | rs6497730 |  |  |
| GABA system | *GABRA2* | rs279858 | Associated with alcohol use disorder and nicotine dependence. | [^90-92^](#_ENREF_90) |
|  | *GABRG2* | rs211014 | Associated with susceptibility to heroin addiction. |  |
| Dopamine system | *DRD2* | rs1079597 | Associated with susceptibility to heroin addiction. |  |
|  | *ANKK1* | rs1800497 | Associated with susceptibility to opiate addiction, relapse, and methadone treatment outcomes. |  |
|  | *DRD4* | VNTR | Long-repeat alleles more prevalent in opioid abusers and associated with novelty seeking traits. | [^102-105^](#_ENREF_102) |
| Other variants | *COMT* | rs4680 | Associated with susceptibility to opiate addiction. |  |
|  | *BDNF* | rs6265 | Associated with susceptibility to heroin addiction and age of onset of substance abuse. | [^108-112^](#_ENREF_108) |
|  | *NGFB* | rs2239622 | Associated with methadone dose requirement in heroin abusers. | [^113^](#_ENREF_113) |
|  | *CSNK1E* | rs135745 | Associated with susceptibility to heroin addiction and amount of self-injected heroin. | [^114^](#_ENREF_114) |
|  | *AVPR1R* | rs1587097 | Associated with susceptibility to heroin addiction. | [^115^](#_ENREF_115) |
|  | *MAOA* | rs1137070 | Associated with susceptibility to heroin addiction and changes in gray matter. | [^116^](#_ENREF_116) |
|  | *ZNF804A* | rs7597593 | Associated with susceptibility to heroin addiction, neurocognitive changes, and changes in gray matter. |  |
|  |  | rs1344706 |  |  |

Table S4. List of 131 significantly different reward network connections between heroin abusers and healthy controls.

|  | **Heroin abusers (*n* = 78)** | **Healthy controls (*n* = 79)** | ***t*** |
| --- | --- | --- | --- |
| AMYG_L-VTA_L' | 0.3126 ± 0.0183 | 0.3556 ± 0.0179 | 14.8853 |
| AMYG_L-VTA_R' | 0.3151 ± 0.0181 | 0.3522 ± 0.0174 | 13.0753 |
| VStr_L-VTA_L' | 0.3312 ± 0.0223 | 0.3745 ± 0.0197 | 12.9012 |
| OFC_L-VTA_L' | 0.2981 ± 0.0208 | 0.3416 ± 0.0215 | 12.8932 |
| INS_L-VTA_L' | 0.2949 ± 0.0182 | 0.3307 ± 0.0175 | 12.5501 |
| OFC_L-VTA_R' | 0.3020 ± 0.0214 | 0.3413 ± 0.0212 | 11.5116 |
| VStr_R-VTA_L' | 0.3347 ± 0.0226 | 0.3727 ± 0.0194 | 11.3391 |
| INS_L-CAU_L' | 0.2959 ± 0.0185 | 0.3281 ± 0.0172 | 11.3014 |
| ACC_L-INS_L' | 0.2795 ± 0.0169 | 0.3092 ± 0.0164 | 11.1813 |
| ACC_R-INS_L' | 0.2796 ± 0.0172 | 0.3098 ± 0.0167 | 11.1646 |
| ACC_L-ACC_R' | 0.2729 ± 0.0194 | 0.3071 ± 0.0193 | 11.0396 |
| INS_L-VTA_R' | 0.2966 ± 0.0183 | 0.3280 ± 0.0175 | 11.0137 |
| OFC_L-CAU_L' | 0.2973 ± 0.0201 | 0.3318 ± 0.0194 | 10.9682 |
| ACC_L-OFC_L' | 0.2804 ± 0.0174 | 0.3099 ± 0.0174 | 10.6357 |
| VStr_L-CAU_L' | 0.3175 ± 0.0212 | 0.3501 ± 0.0176 | 10.5033 |
| ACC_R-VStr_L' | 0.2846 ± 0.0187 | 0.3150 ± 0.0178 | 10.4368 |
| ACC_R-OFC_L' | 0.2805 ± 0.0178 | 0.3096 ± 0.0178 | 10.2500 |
| VStr_L-VTA_R' | 0.3382 ± 0.0235 | 0.3733 ± 0.0193 | 10.2268 |
| ACC_L-VStr_L' | 0.2851 ± 0.0180 | 0.3138 ± 0.0176 | 10.0984 |
| ACC_R-VTA_L' | 0.2874 ± 0.0184 | 0.3166 ± 0.0180 | 10.0681 |
| INS_L-PUT_L' | 0.2941 ± 0.0178 | 0.3220 ± 0.0171 | 9.9908 |
| HIP_L-VTA_L' | 0.3066 ± 0.0171 | 0.3329 ± 0.0167 | 9.7586 |
| INS_L-VStr_L' | 0.3021 ± 0.0183 | 0.3305 ± 0.0182 | 9.7543 |
| INS_L-PAL_L' | 0.2928 ± 0.0176 | 0.3200 ± 0.0173 | 9.7283 |
| AMYG_L-PUT_L' | 0.2964 ± 0.0179 | 0.3230 ± 0.0170 | 9.5648 |
| OFC_L-PUT_L' | 0.2895 ± 0.0181 | 0.3170 ± 0.0181 | 9.5162 |
| ACC_L-VTA_L' | 0.2882 ± 0.0179 | 0.3149 ± 0.0176 | 9.4443 |
| AMYG_L-CAU_L' | 0.3074 ± 0.0193 | 0.3349 ± 0.0171 | 9.4238 |
| ACC_R-VStr_R' | 0.2844 ± 0.0190 | 0.3123 ± 0.0182 | 9.3800 |
| ACC_R-VTA_R' | 0.2885 ± 0.0186 | 0.3158 ± 0.0182 | 9.2987 |
| ACC_L-VStr_R' | 0.2855 ± 0.0183 | 0.3122 ± 0.0177 | 9.2952 |
| AMYG_L-PAL_L' | 0.2986 ± 0.0178 | 0.3244 ± 0.0170 | 9.2712 |
| VStr_L-PUT_L' | 0.2979 ± 0.0180 | 0.3240 ± 0.0173 | 9.2614 |
| DLPFC_L-VTA_L' | 0.2523 ± 0.0244 | 0.2864 ± 0.0218 | 9.2451 |
| INS_L-THA_L' | 0.2923 ± 0.0166 | 0.3164 ± 0.0161 | 9.2273 |
| OFC_L-PAL_L' | 0.2904 ± 0.0180 | 0.3169 ± 0.0182 | 9.1776 |
| ACC_R-AMYG_L' | 0.2868 ± 0.0173 | 0.3116 ± 0.0166 | 9.1479 |
| INS_L-AMYG_L' | 0.2998 ± 0.0174 | 0.3251 ± 0.0176 | 9.0652 |
| ACC_R-PAL_L' | 0.2821 ± 0.0171 | 0.3067 ± 0.0170 | 9.0123 |
| VStr_R-VTA_R' | 0.3412 ± 0.0237 | 0.3720 ± 0.0194 | 8.9030 |
| OFC_L-THA_L' | 0.2916 ± 0.0171 | 0.3156 ± 0.0172 | 8.7562 |
| INS_L-VStr_R' | 0.3012 ± 0.0183 | 0.3265 ± 0.0180 | 8.7503 |
| VStr_L-PAL_L' | 0.3002 ± 0.0183 | 0.3250 ± 0.0174 | 8.7302 |
| ACC_L-VTA_R' | 0.2904 ± 0.0180 | 0.3153 ± 0.0178 | 8.7142 |
| ACC_R-PUT_L' | 0.2832 ± 0.0168 | 0.3064 ± 0.0166 | 8.7070 |
| AMYG_R-VTA_L' | 0.3141 ± 0.0176 | 0.3383 ± 0.0174 | 8.6674 |
| ACC_L-AMYG_L' | 0.2875 ± 0.0170 | 0.3106 ± 0.0164 | 8.6571 |
| AMYG_R-VTA_R' | 0.3165 ± 0.0179 | 0.3410 ± 0.0177 | 8.5852 |
| ACC_L-PAL_L' | 0.2829 ± 0.0168 | 0.3057 ± 0.0168 | 8.5137 |
| INS_L-OFC_L' | 0.2973 ± 0.0177 | 0.3225 ± 0.0192 | 8.5124 |
| VStr_R-CAU_L' | 0.3194 ± 0.0215 | 0.3459 ± 0.0179 | 8.4251 |
| ACC_L-PUT_L' | 0.2836 ± 0.0166 | 0.3057 ± 0.0164 | 8.3812 |
| OFC_L-AMYG_L' | 0.3056 ± 0.0188 | 0.3326 ± 0.0214 | 8.3778 |
| DLPFC_L-VTA_R' | 0.2536 ± 0.0248 | 0.2850 ± 0.0221 | 8.3683 |
| ACC_R-CAU_L' | 0.2916 ± 0.0176 | 0.3146 ± 0.0173 | 8.2567 |
| INS_L-CAU_R' | 0.2978 ± 0.0190 | 0.3218 ± 0.0176 | 8.2439 |
| HIP_L-PUT_L' | 0.2941 ± 0.0172 | 0.3160 ± 0.0164 | 8.1535 |
| VTA_L-PAL_L' | 0.3039 ± 0.0174 | 0.3263 ± 0.0172 | 8.1162 |
| HIP_L-VTA_R' | 0.3075 ± 0.0171 | 0.3291 ± 0.0164 | 8.0661 |
| VTA_L-PUT_L' | 0.3031 ± 0.0173 | 0.3251 ± 0.0171 | 7.9938 |
| VStr_L-THA_L' | 0.3016 ± 0.0169 | 0.3223 ± 0.0160 | 7.9036 |
| OFC_L-CAU_R' | 0.3044 ± 0.0211 | 0.3299 ± 0.0194 | 7.9028 |
| VStr_R-PUT_L' | 0.2984 ± 0.0182 | 0.3206 ± 0.0173 | 7.8386 |
| ACC_R-CAU_R' | 0.2924 ± 0.0182 | 0.3145 ± 0.0176 | 7.7345 |
| ACC_L-CAU_L' | 0.2927 ± 0.0172 | 0.3136 ± 0.0170 | 7.6495 |
| ACC_R-THA_L' | 0.2870 ± 0.0158 | 0.3061 ± 0.0155 | 7.6495 |
| ACC_L-CAU_R' | 0.2926 ± 0.0178 | 0.3140 ± 0.0173 | 7.6418 |
| PAL_L-PUT_L' | 0.3007 ± 0.0174 | 0.3217 ± 0.0171 | 7.6159 |
| OFC_L-VStr_L' | 0.3108 ± 0.0215 | 0.3383 ± 0.0237 | 7.6029 |
| HIP_L-CAU_L' | 0.3024 ± 0.0173 | 0.3227 ± 0.0162 | 7.6002 |
| HIP_L-PAL_L' | 0.2962 ± 0.0169 | 0.3162 ± 0.0162 | 7.5951 |
| AMYG_L-THA_L' | 0.2994 ± 0.0167 | 0.3192 ± 0.0161 | 7.5893 |
| VStr_R-PAL_L' | 0.3008 ± 0.0183 | 0.3221 ± 0.0175 | 7.4717 |
| ACC_R-HIP_L' | 0.2875 ± 0.0163 | 0.3066 ± 0.0157 | 7.4563 |
| VTA_R-PAL_L' | 0.3036 ± 0.0175 | 0.3242 ± 0.0175 | 7.3684 |
| VTA_R-PUT_L' | 0.3022 ± 0.0174 | 0.3224 ± 0.0173 | 7.3093 |
| CAU_L-PUT_L' | 0.3023 ± 0.0174 | 0.3223 ± 0.0169 | 7.3045 |
| INS_L-HIP_L' | 0.2964 ± 0.0165 | 0.3158 ± 0.0167 | 7.2951 |
| ACC_R-AMYG_R' | 0.2886 ± 0.0172 | 0.3084 ± 0.0173 | 7.1639 |
| CAU_L-PAL_L' | 0.3035 ± 0.0176 | 0.3233 ± 0.0170 | 7.1473 |
| ACC_L-HIP_L' | 0.2881 ± 0.0160 | 0.3058 ± 0.0155 | 7.0755 |
| ACC_L-THA_L' | 0.2879 ± 0.0157 | 0.3053 ± 0.0154 | 7.0338 |
| DLPFC_L-CAU_L' | 0.2635 ± 0.0227 | 0.2872 ± 0.0202 | 6.9127 |
| PUT_L-THA_L' | 0.2981 ± 0.0165 | 0.3160 ± 0.0163 | 6.8261 |
| HIP_L-THA_L' | 0.2981 ± 0.0165 | 0.3156 ± 0.0159 | 6.7348 |
| ACC_L-AMYG_R' | 0.2891 ± 0.0168 | 0.3072 ± 0.0169 | 6.7133 |
| VStr_R-THA_L' | 0.3013 ± 0.0171 | 0.3187 ± 0.0160 | 6.6136 |
| VStr_R-CAU_R' | 0.3296 ± 0.0231 | 0.3513 ± 0.0179 | 6.5931 |
| INS_L-AMYG_R' | 0.2965 ± 0.0170 | 0.3145 ± 0.0174 | 6.5600 |
| PAL_L-THA_L' | 0.2980 ± 0.0164 | 0.3150 ± 0.0161 | 6.5399 |
| DLPFC_R-VTA_R' | 0.2521 ± 0.0255 | 0.2768 ± 0.0229 | 6.4024 |
| DLPFC_R-VTA_L' | 0.2583 ± 0.0251 | 0.2824 ± 0.0224 | 6.3359 |
| VStr_L-CAU_R' | 0.3260 ± 0.0231 | 0.3467 ± 0.0179 | 6.2785 |
| AMYG_L-VStr_L' | 0.3312 ± 0.0194 | 0.3505 ± 0.0196 | 6.1972 |
| CAU_L-THA_L' | 0.3071 ± 0.0166 | 0.3230 ± 0.0159 | 6.1381 |
| OFC_L-VStr_R' | 0.3129 ± 0.0219 | 0.3352 ± 0.0238 | 6.0943 |
| AMYG_L-CAU_R' | 0.3157 ± 0.0198 | 0.3337 ± 0.0174 | 6.0481 |
| AMYG_R-PUT_L' | 0.2956 ± 0.0176 | 0.3124 ± 0.0171 | 6.0455 |
| DLPFC_L-PUT_L' | 0.2696 ± 0.0193 | 0.2876 ± 0.0182 | 6.0053 |
| HIP_R-VTA_R' | 0.3085 ± 0.0164 | 0.3241 ± 0.0165 | 5.9240 |
| DLPFC_R-CAU_R' | 0.2681 ± 0.0234 | 0.2871 ± 0.0205 | 5.8900 |
| OFC_R-VTA_L' | 0.3061 ± 0.0215 | 0.3259 ± 0.0206 | 5.8875 |
| VTA_L-CAU_L' | 0.3294 ± 0.0190 | 0.3465 ± 0.0177 | 5.8446 |
| CAU_R-PAL_L' | 0.3016 ± 0.0179 | 0.3177 ± 0.0175 | 5.6895 |
| AMYG_R-PAL_L' | 0.2985 ± 0.0175 | 0.3141 ± 0.0170 | 5.6706 |
| VTA_L-THA_L' | 0.3075 ± 0.0164 | 0.3221 ± 0.0158 | 5.6661 |
| DLPFC_L-PAL_L' | 0.2688 ± 0.0198 | 0.2862 ± 0.0189 | 5.6215 |
| HIP_R-VTA_L' | 0.3069 ± 0.0163 | 0.3215 ± 0.0164 | 5.5759 |
| VTA_R-THA_L' | 0.3059 ± 0.0166 | 0.3203 ± 0.0159 | 5.5485 |
| CAU_R-PUT_L' | 0.2998 ± 0.0178 | 0.3152 ± 0.0173 | 5.5198 |
| AMYG_R-CAU_L' | 0.3113 ± 0.0188 | 0.3271 ± 0.0175 | 5.4260 |
| DLPFC_L-CAU_R' | 0.2626 ± 0.0237 | 0.2837 ± 0.0211 | 5.3967 |
| INS_L-HIP_R' | 0.2944 ± 0.0166 | 0.3087 ± 0.0168 | 5.3696 |
| ACC_R-DLPFC_L' | 0.2593 ± 0.0212 | 0.2772 ± 0.0211 | 5.2845 |
| INS_L-THA_R' | 0.2919 ± 0.0170 | 0.3059 ± 0.0163 | 5.2520 |
| INS_L-OFC_R' | 0.2922 ± 0.0185 | 0.3077 ± 0.0193 | 5.1407 |
| HIP_L-AMYG_L' | 0.3146 ± 0.0168 | 0.3287 ± 0.0174 | 5.1403 |
| HIP_L-OFC_L' | 0.3013 ± 0.0176 | 0.3165 ± 0.0195 | 5.1269 |
| ACC_R-HIP_R' | 0.2887 ± 0.0161 | 0.3018 ± 0.0160 | 5.1024 |
| DLPFC_L-THA_L' | 0.2709 ± 0.0179 | 0.2851 ± 0.0170 | 5.0851 |
| ACC_L-THA_R' | 0.2883 ± 0.0160 | 0.3011 ± 0.0156 | 5.0792 |
| OFC_L-AMYG_R' | 0.3047 ± 0.0185 | 0.3204 ± 0.0203 | 5.0560 |
| HIP_R-PAL_L' | 0.2959 ± 0.0168 | 0.3093 ± 0.0165 | 5.0413 |
| DLPFC_R-INS_L' | 0.2676 ± 0.0197 | 0.2832 ± 0.0192 | 5.0239 |
| ACC_R-PAL_R' | 0.2870 ± 0.0171 | 0.3008 ± 0.0175 | 5.0115 |
| ACC_R-THA_R' | 0.2884 ± 0.0161 | 0.3010 ± 0.0157 | 4.9861 |
| HIP_R-PUT_L' | 0.2948 ± 0.0171 | 0.3081 ± 0.0167 | 4.9449 |
| DLPFC_R-CAU_L' | 0.2685 ± 0.0230 | 0.2857 ± 0.0209 | 4.9203 |
| CAU_R-THA_L' | 0.3020 ± 0.0169 | 0.3148 ± 0.0160 | 4.8855 |
| ACC_L-PAL_R' | 0.2871 ± 0.0170 | 0.3004 ± 0.0174 | 4.8719 |
| OFC_R-PUT_L' | 0.2918 ± 0.0183 | 0.3059 ± 0.0182 | 4.8483 |

The data are expressed as mean ± standard deviation. L' and R' represent the left and right hemispheres, respectively. The abbreviation for each brain area are shown in Table S2.

Table S5. Mediation analysis for *GABRA2* rs279858, reward network and MoCA.

| X | M | Y | Healthy controls | | | Heroin abusers | | |
| --- | --- | --- | --- | --- | --- | --- | --- | --- |
|  |  |  | Indirect effect | BootLLCI | BootULCI | Indirect effect | BootLLCI | BootULCI |
| *GABRA2* rs279858 | The mean strength of control subnetwork | Cognition evaluated by the MoCA | -0.3014 | -0.8935 | .0263 | -0.0043 | -0.1941 | 0.0912 |
|  | The mean strength of driving subnetwork |  | -0.3468 | -0.9058 | -0.0316 | -0.0077 | -0.2365 | 0.0884 |
|  | The mean strength of BTN-connections |  | -0.3346 | -0.9215 | -0.0018 | -0.0064 | -0.2156 | 0.0850 |
|  | The mean strength of Diff-connections |  | -0.3289 | -0.8763 | -0.0007 | -0.0128 | -0.2640 | 0.0721 |

*BootLLCI* bootstrap lower limit confidence interval, *BootULCI* bootstrap upper limit confidence interval.


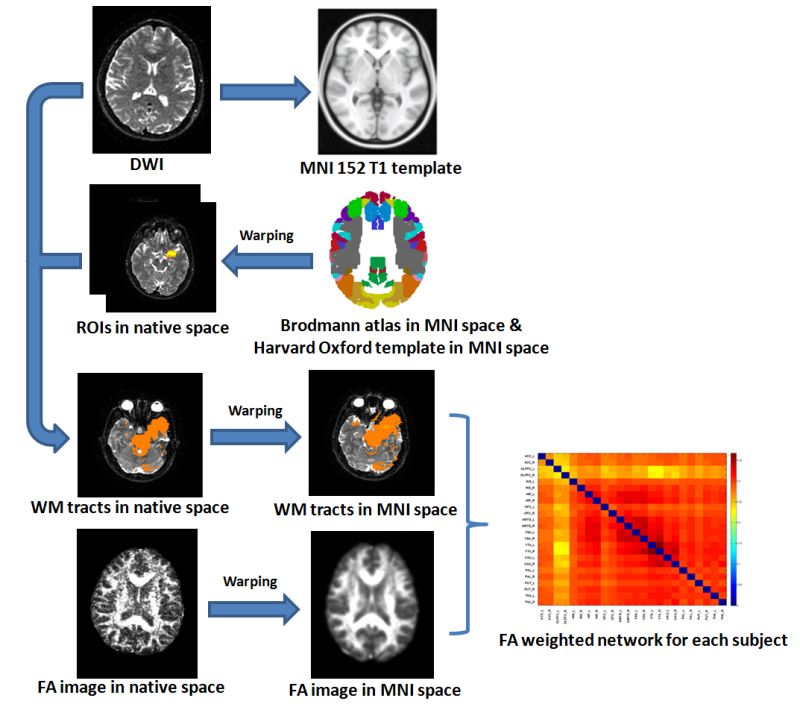


**Figure S1.** **Flow chart of the network analysis.** Reward-related ROIs were defined using the Brodmann atlas and Harvard Oxford template in MNI152 space. These ROIs were warped to native diffusion image space for measuring structure connectivity between the ROIs. All measurements in the native diffusion image space were warped to the MNI space for defining white matter (WM) streamline regions using one sample t-test (*p* < 0.05) for heroin abusers and healthy controls respectively. The connective strength of each edge was defined as the average FA values of all voxels that were included in the streamline regions.


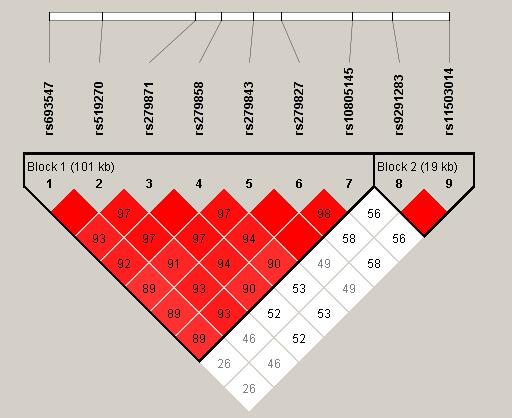


a

b


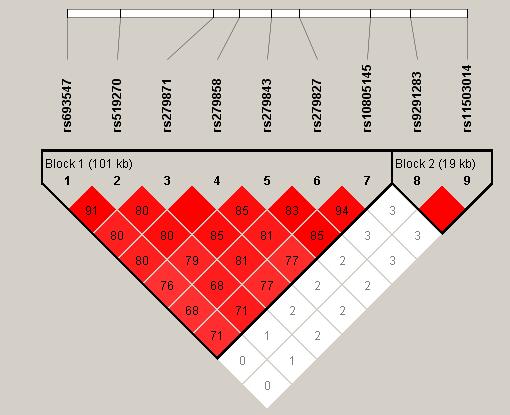


**Figure S2. Linkage disequilibrium (LD) map between markers genotyped in *GABRA2* in our imaging samples.** The LD structure between marker pairs is indicated by the shaded matrices. The figure was generated using HaploView 4.1. (a) D’ value. (b) *r^2^* value.


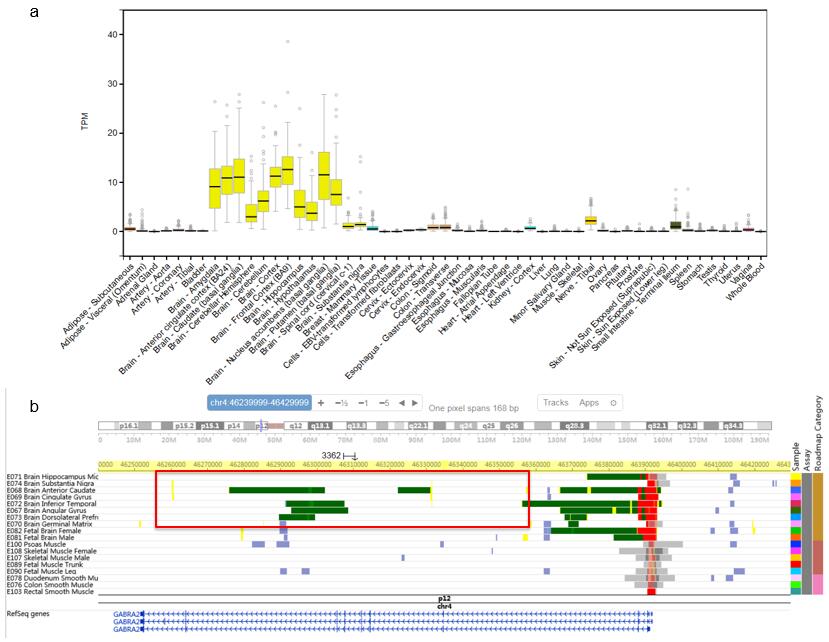
**Figure S3. Functional analysis for rs279858 LD-block and GABRA2.** (a) gene expression of *GABRA2* in different tissues obtained ­­from GTEx[^119^](#_ENREF_119). (b) chromatin state of *GABRA2* from the Roadmap Epigenomics Project[^120^](#_ENREF_120) viewed using WashU EpiGenome Browser. 10 brain tissues with 8 muscle tissues for comparison. Red box denotes the rs279858 related LD-block.

**References**

1. Luis CA, Keegan AP, Mullan M. Cross validation of the Montreal Cognitive Assessment in community dwelling older adults residing in the Southeastern US. *International journal of geriatric psychiatry* 2009; **24**(2)**:** 197-201.

2. Patton JH, Stanford MS, Barratt ES. Factor structure of the Barratt impulsiveness scale. *Journal of clinical psychology* 1995; **51**(6)**:** 768-774.

3. Bechara A, Damasio AR, Damasio H, Anderson SW. Insensitivity to future consequences following damage to human prefrontal cortex. *Cognition* 1994; **50**(1-3)**:** 7-15.

4. Bechara A, Tranel D, Damasio H. Characterization of the decision-making deficit of patients with ventromedial prefrontal cortex lesions. *Brain : a journal of neurology* 2000; **123 ( Pt 11):** 2189-2202.

5. Bickel WK, DeGrandpre RJ, Higgins ST. Behavioral economics: a novel experimental approach to the study of drug dependence. *Drug and alcohol dependence* 1993; **33**(2)**:** 173-192.

6. Li Q, *et al*. Predicting subsequent relapse by drug-related cue-induced brain activation in heroin addiction: an event-related functional magnetic resonance imaging study. *Addiction biology* 2015; **20**(5)**:** 968-978.

7. Li Q, *et al*. Craving correlates with mesolimbic responses to heroin-related cues in short-term abstinence from heroin: an event-related fMRI study. *Brain research* 2012; **1469:** 63-72.

8. Li Q, *et al*. Abnormal function of the posterior cingulate cortex in heroin addicted users during resting-state and drug-cue stimulation task. *Chinese medical journal* 2013; **126**(4)**:** 734-739.

9. Artiges E, *et al*. Exposure to smoking cues during an emotion recognition task can modulate limbic fMRI activation in cigarette smokers. *Addiction biology* 2009; **14**(4)**:** 469-477.

10. Nestor L, McCabe E, Jones J, Clancy L, Garavan H. Differences in "bottom-up" and "top-down" neural activity in current and former cigarette smokers: Evidence for neural substrates which may promote nicotine abstinence through increased cognitive control. *NeuroImage* 2011; **56**(4)**:** 2258-2275.

11. Tabatabaei-Jafari H, *et al*. Patterns of brain activation during craving in heroin dependents successfully treated by methadone maintenance and abstinence-based treatments. *Journal of addiction medicine* 2014; **8**(2)**:** 123-129.

12. Canterberry M, Peltier MR, Brady KT, Hanlon CA. Attenuated neural response to emotional cues in cocaine-dependence: a preliminary analysis of gender differences. *The American journal of drug and alcohol abuse* 2016**:** 1-10.

13. Goudriaan AE, Veltman DJ, van den Brink W, Dom G, Schmaal L. Neurophysiological effects of modafinil on cue-exposure in cocaine dependence: a randomized placebo-controlled cross-over study using pharmacological fMRI. *Addictive behaviors* 2013; **38**(2)**:** 1509-1517.

14. Goldstein RZ, *et al*. Anterior cingulate cortex hypoactivations to an emotionally salient task in cocaine addiction. *Proceedings of the National Academy of Sciences of the United States of America* 2009; **106**(23)**:** 9453-9458.

15. Goldstein RZ, *et al*. Dopaminergic response to drug words in cocaine addiction. *The Journal of neuroscience : the official journal of the Society for Neuroscience* 2009; **29**(18)**:** 6001-6006.

16. Goldstein RZ, *et al*. Oral methylphenidate normalizes cingulate activity in cocaine addiction during a salient cognitive task. *Proceedings of the National Academy of Sciences of the United States of America* 2010; **107**(38)**:** 16667-16672.

17. Potenza MN, *et al*. Neural correlates of stress-induced and cue-induced drug craving: influences of sex and cocaine dependence. *The American journal of psychiatry* 2012; **169**(4)**:** 406-414.

18. Luijten M, *et al*. Brain activation associated with attentional bias in smokers is modulated by a dopamine antagonist. *Neuropsychopharmacology : official publication of the American College of Neuropsychopharmacology* 2012; **37**(13)**:** 2772-2779.

19. Luijten M, *et al*. Neurobiological substrate of smoking-related attentional bias. *NeuroImage* 2011; **54**(3)**:** 2374-2381.

20. Zhang X, *et al*. Anatomical differences and network characteristics underlying smoking cue reactivity. *NeuroImage* 2011; **54**(1)**:** 131-141.

21. Fryer SL, *et al*. Differential brain response to alcohol cue distractors across stages of alcohol dependence. *Biological psychology* 2013; **92**(2)**:** 282-291.

22. Tapert SF, Brown GG, Baratta MV, Brown SA. fMRI BOLD response to alcohol stimuli in alcohol dependent young women. *Addictive behaviors* 2004; **29**(1)**:** 33-50.

23. Wesley MJ, Hanlon CA, Porrino LJ. Poor decision-making by chronic marijuana users is associated with decreased functional responsiveness to negative consequences. *Psychiatry research* 2011; **191**(1)**:** 51-59.

24. Filbey FM, *et al*. fMRI study of neural sensitization to hedonic stimuli in long-term, daily cannabis users. *Human brain mapping* 2016.

25. Gowin JL, *et al*. Altered cingulate and insular cortex activation during risk-taking in methamphetamine dependence: losses lose impact. *Addiction (Abingdon, England)* 2014; **109**(2)**:** 237-247.

26. Monterosso JR, *et al*. Frontoparietal cortical activity of methamphetamine-dependent and comparison subjects performing a delay discounting task. *Human brain mapping* 2007; **28**(5)**:** 383-393.

27. Wrase J, *et al*. Development of alcohol-associated cues and cue-induced brain activation in alcoholics. *European psychiatry : the journal of the Association of European Psychiatrists* 2002; **17**(5)**:** 287-291.

28. Wexler BE, *et al*. Functional magnetic resonance imaging of cocaine craving. *The American journal of psychiatry* 2001; **158**(1)**:** 86-95.

29. Yang Z, *et al*. Dynamic neural responses to cue-reactivity paradigms in heroin-dependent users: an fMRI study. *Human brain mapping* 2009; **30**(3)**:** 766-775.

30. Yalachkov Y, Kaiser J, Naumer MJ. Brain regions related to tool use and action knowledge reflect nicotine dependence. *The Journal of neuroscience : the official journal of the Society for Neuroscience* 2009; **29**(15)**:** 4922-4929.

31. Yip SW, *et al*. Anticipatory reward processing among cocaine-dependent individuals with and without concurrent methadone-maintenance treatment: Relationship to treatment response. *Drug and alcohol dependence* 2016.

32. Wesley MJ, *et al*. Choosing Money over Drugs: The Neural Underpinnings of Difficult Choice in Chronic Cocaine Users. *Journal of addiction* 2014; **2014:** 189853.

33. Lee E, *et al*. Neural evidence for emotional involvement in pathological alcohol craving. *Alcohol and alcoholism (Oxford, Oxfordshire)* 2013; **48**(3)**:** 288-294.

34. George MS, *et al*. Activation of prefrontal cortex and anterior thalamus in alcoholic subjects on exposure to alcohol-specific cues. *Archives of general psychiatry* 2001; **58**(4)**:** 345-352.

35. Gradin VB, Baldacchino A, Balfour D, Matthews K, Steele JD. Abnormal brain activity during a reward and loss task in opiate-dependent patients receiving methadone maintenance therapy. *Neuropsychopharmacology : official publication of the American College of Neuropsychopharmacology* 2014; **39**(4)**:** 885-894.

36. Jia Z, *et al*. An initial study of neural responses to monetary incentives as related to treatment outcome in cocaine dependence. *Biological psychiatry* 2011; **70**(6)**:** 553-560.

37. Kobiella A, *et al*. Acute and chronic nicotine effects on behaviour and brain activation during intertemporal decision making. *Addiction biology* 2014; **19**(5)**:** 918-930.

38. Ihssen N, Cox WM, Wiggett A, Fadardi JS, Linden DE. Differentiating heavy from light drinkers by neural responses to visual alcohol cues and other motivational stimuli. *Cerebral cortex (New York, NY : 1991)* 2011; **21**(6)**:** 1408-1415.

39. Cousijn J, *et al*. Individual differences in decision making and reward processing predict changes in cannabis use: a prospective functional magnetic resonance imaging study. *Addiction biology* 2013; **18**(6)**:** 1013-1023.

40. Nestor L, Hester R, Garavan H. Increased ventral striatal BOLD activity during non-drug reward anticipation in cannabis users. *NeuroImage* 2010; **49**(1)**:** 1133-1143.

41. May AC, Stewart JL, Migliorini R, Tapert SF, Paulus MP. Methamphetamine dependent individuals show attenuated brain response to pleasant interoceptive stimuli. *Drug and alcohol dependence* 2013; **131**(3)**:** 238-246.

42. Migliorini R, Stewart JL, May AC, Tapert SF, Paulus MP. What do you feel? Adolescent drug and alcohol users show altered brain response to pleasant interoceptive stimuli. *Drug and alcohol dependence* 2013; **133**(2)**:** 661-668.

43. Stewart JL, May AC, Tapert SF, Paulus MP. Hyperactivation to pleasant interoceptive stimuli characterizes the transition to stimulant addiction. *Drug and alcohol dependence* 2015; **154:** 264-270.

44. Tobler PN, *et al*. Shared neural basis of social and non-social reward deficits in chronic cocaine users. *Social cognitive and affective neuroscience* 2016; **11**(6)**:** 1017-1025.

45. Young KA, *et al*. Nipping cue reactivity in the bud: baclofen prevents limbic activation elicited by subliminal drug cues. *The Journal of neuroscience : the official journal of the Society for Neuroscience* 2014; **34**(14)**:** 5038-5043.

46. Ames SL, *et al*. Functional imaging of an alcohol-Implicit Association Test (IAT). *Addiction biology* 2014; **19**(3)**:** 467-481.

47. Hermann D, *et al*. Blockade of cue-induced brain activation of abstinent alcoholics by a single administration of amisulpride as measured with fMRI. *Alcoholism, clinical and experimental research* 2006; **30**(8)**:** 1349-1354.

48. Konova AB, *et al*. Converging effects of cocaine addiction and sex on neural responses to monetary rewards. *Psychiatry research* 2016; **248:** 110-118.

49. Rubinstein ML, Luks TL, Dryden WY, Rait MA, Simpson GV. Adolescent smokers show decreased brain responses to pleasurable food images compared with nonsmokers. *Nicotine & tobacco research : official journal of the Society for Research on Nicotine and Tobacco* 2011; **13**(8)**:** 751-755.

50. Ames SL, *et al*. Functional imaging of implicit marijuana associations during performance on an Implicit Association Test (IAT). *Behavioural brain research* 2013; **256:** 494-502.

51. Heinz A, *et al*. Brain activation elicited by affectively positive stimuli is associated with a lower risk of relapse in detoxified alcoholic subjects. *Alcoholism, clinical and experimental research* 2007; **31**(7)**:** 1138-1147.

52. Yalachkov Y, Kaiser J, Gorres A, Seehaus A, Naumer MJ. Sensory modality of smoking cues modulates neural cue reactivity. *Psychopharmacology* 2013; **225**(2)**:** 461-471.

53. Park MS, *et al*. Brain substrates of craving to alcohol cues in subjects with alcohol use disorder. *Alcohol and alcoholism (Oxford, Oxfordshire)* 2007; **42**(5)**:** 417-422.

54. Tapert SF, *et al*. Neural response to alcohol stimuli in adolescents with alcohol use disorder. *Archives of general psychiatry* 2003; **60**(7)**:** 727-735.

55. Braus DF, *et al*. Alcohol-associated stimuli activate the ventral striatum in abstinent alcoholics. *J Neural Transm (Vienna)* 2001; **108**(7)**:** 887-894.

56. Asensio S, *et al*. Altered neural response of the appetitive emotional system in cocaine addiction: an fMRI Study. *Addiction biology* 2010; **15**(4)**:** 504-516.

57. Tau GZ, *et al*. Neural correlates of reward-based spatial learning in persons with cocaine dependence. *Neuropsychopharmacology : official publication of the American College of Neuropsychopharmacology* 2014; **39**(3)**:** 545-555.

58. Vaquero L, *et al*. Cocaine addiction is associated with abnormal prefrontal function, increased striatal connectivity and sensitivity to monetary incentives, and decreased connectivity outside the human reward circuit. *Addiction biology* 2016.

59. Bell RP, Garavan H, Foxe JJ. Neural correlates of craving and impulsivity in abstinent former cocaine users: Towards biomarkers of relapse risk. *Neuropharmacology* 2014; **85:** 461-470.

60. Luo S, Ainslie G, Giragosian L, Monterosso JR. Striatal hyposensitivity to delayed rewards among cigarette smokers. *Drug and alcohol dependence* 2011; **116**(1-3)**:** 18-23.

61. Beck A, *et al*. Ventral striatal activation during reward anticipation correlates with impulsivity in alcoholics. *Biological psychiatry* 2009; **66**(8)**:** 734-742.

62. Beck A, *et al*. Effect of brain structure, brain function, and brain connectivity on relapse in alcohol-dependent patients. *Archives of general psychiatry* 2012; **69**(8)**:** 842-852.

63. Vollstadt-Klein S, *et al*. Initial, habitual and compulsive alcohol use is characterized by a shift of cue processing from ventral to dorsal striatum. *Addiction (Abingdon, England)* 2010; **105**(10)**:** 1741-1749.

64. Wrase J, *et al*. Dysfunction of reward processing correlates with alcohol craving in detoxified alcoholics. *NeuroImage* 2007; **35**(2)**:** 787-794.

65. Malcolm R, *et al*. Regional Brain Activity in Abstinent Methamphetamine Dependent Males Following Cue Exposure. *Journal of drug abuse* 2016; **2**(1).

66. van Hell HH, *et al*. Chronic effects of cannabis use on the human reward system: an fMRI study. *European neuropsychopharmacology : the journal of the European College of Neuropsychopharmacology* 2010; **20**(3)**:** 153-163.

67. Zijlstra F, Veltman DJ, Booij J, van den Brink W, Franken IH. Neurobiological substrates of cue-elicited craving and anhedonia in recently abstinent opioid-dependent males. *Drug and alcohol dependence* 2009; **99**(1-3)**:** 183-192.

68. Schneider F, *et al*. Subcortical correlates of craving in recently abstinent alcoholic patients. *The American journal of psychiatry* 2001; **158**(7)**:** 1075-1083.

69. Rose EJ, *et al*. Acute nicotine differentially impacts anticipatory valence- and magnitude-related striatal activity. *Biological psychiatry* 2013; **73**(3)**:** 280-288.

70. Filbey FM, Dunlop J, Myers US. Neural effects of positive and negative incentives during marijuana withdrawal. *PloS one* 2013; **8**(5)**:** e61470.

71. D'Ardenne K, McClure SM, Nystrom LE, Cohen JD. BOLD responses reflecting dopaminergic signals in the human ventral tegmental area. *Science (New York, NY)* 2008; **319**(5867)**:** 1264-1267.

72. Mayer P, *et al*. Association between a delta opioid receptor gene polymorphism and heroin dependence in man. *Neuroreport* 1997; **8**(11)**:** 2547-2550.

73. Yuferov V, *et al*. Redefinition of the human kappa opioid receptor gene (OPRK1) structure and association of haplotypes with opiate addiction. *Pharmacogenetics* 2004; **14**(12)**:** 793-804.

74. Gerra G, *et al*. Human kappa opioid receptor gene (OPRK1) polymorphism is associated with opiate addiction. *American journal of medical genetics Part B, Neuropsychiatric genetics : the official publication of the International Society of Psychiatric Genetics* 2007; **144b**(6)**:** 771-775.

75. Luo X, *et al*. ADH4 gene variation is associated with alcohol and drug dependence: results from family controlled and population-structured association studies. *Pharmacogenetics and genomics* 2005; **15**(11)**:** 755-768.

76. Bond C, *et al*. Single-nucleotide polymorphism in the human mu opioid receptor gene alters beta-endorphin binding and activity: possible implications for opiate addiction. *Proceedings of the National Academy of Sciences of the United States of America* 1998; **95**(16)**:** 9608-9613.

77. Bart G, *et al*. Substantial attributable risk related to a functional mu-opioid receptor gene polymorphism in association with heroin addiction in central Sweden. *Molecular psychiatry* 2004; **9**(6)**:** 547-549.

78. Nagaya D, Ramanathan S, Ravichandran M, Navaratnam V. A118G mu opioid receptor polymorphism among drug addicts in Malaysia. *Journal of integrative neuroscience* 2012; **11**(1)**:** 117-122.

79. Kapur S, Sharad S, Singh RA, Gupta AK. A118g polymorphism in mu opioid receptor gene (oprm1): association with opiate addiction in subjects of Indian origin. *Journal of integrative neuroscience* 2007; **6**(4)**:** 511-522.

80. Haerian BS, Haerian MS. OPRM1 rs1799971 polymorphism and opioid dependence: evidence from a meta-analysis. *Pharmacogenomics* 2013; **14**(7)**:** 813-824.

81. Schwantes-An TH, *et al*. Association of the OPRM1 Variant rs1799971 (A118G) with Non-Specific Liability to Substance Dependence in a Collaborative de novo Meta-Analysis of European-Ancestry Cohorts. *Behavior genetics* 2016; **46**(2)**:** 151-169.

82. Gao F, Zhu YS, Wei SG, Li SB, Lai JH. Polymorphism G861C of 5-HT receptor subtype 1B is associated with heroin dependence in Han Chinese. *Biochemical and biophysical research communications* 2011; **412**(3)**:** 450-453.

83. Cao J, LaRocque E, Li D. Associations of the 5-hydroxytryptamine (serotonin) receptor 1B gene (HTR1B) with alcohol, cocaine, and heroin abuse. *American journal of medical genetics Part B, Neuropsychiatric genetics : the official publication of the International Society of Psychiatric Genetics* 2013; **162b**(2)**:** 169-176.

84. Gerra G, *et al*. Association between low-activity serotonin transporter genotype and heroin dependence: behavioral and personality correlates. *American journal of medical genetics Part B, Neuropsychiatric genetics : the official publication of the International Society of Psychiatric Genetics* 2004; **126b**(1)**:** 37-42.

85. Wang TY, *et al*. TPH1 and 5-HTTLPR Genes Specifically Interact in Opiate Dependence but Not in Alcohol Dependence. *European addiction research* 2016; **22**(4)**:** 201-209.

86. Wang TY, *et al*. Association between DRD2, 5-HTTLPR, and ALDH2 genes and specific personality traits in alcohol- and opiate-dependent patients. *Behavioural brain research* 2013; **250:** 285-292.

87. Wu W, Zhu YS, Li SB. Polymorphisms in the glutamate decarboxylase 1 gene associated with heroin dependence. *Biochemical and biophysical research communications* 2012; **422**(1)**:** 91-96.

88. Levran O, *et al*. Heroin addiction in African Americans: a hypothesis-driven association study. *Genes, brain, and behavior* 2009; **8**(5)**:** 531-540.

89. Zhao B, *et al*. Analysis of variations in the glutamate receptor, N-methyl D-aspartate 2A (GRIN2A) gene reveals their relative importance as genetic susceptibility factors for heroin addiction. *PloS one* 2013; **8**(8)**:** e70817.

90. Olfson E, Bierut LJ. Convergence of genome-wide association and candidate gene studies for alcoholism. *Alcoholism, clinical and experimental research* 2012; **36**(12)**:** 2086-2094.

91. Strac DS, *et al*. Association of GABAA receptor alpha2 subunit gene (GABRA2) with alcohol dependence-related aggressive behavior. *Progress in neuro-psychopharmacology & biological psychiatry* 2015; **63:** 119-125.

92. Agrawal A, *et al*. Gamma-aminobutyric acid receptor genes and nicotine dependence: evidence for association from a case-control study. *Addiction (Abingdon, England)* 2008; **103**(6)**:** 1027-1038.

93. Li D, *et al*. Association of gamma-aminobutyric acid A receptor alpha2 gene (GABRA2) with alcohol use disorder. *Neuropsychopharmacology : official publication of the American College of Neuropsychopharmacology* 2014; **39**(4)**:** 907-918.

94. Loh EW, Tang NL, Lee DT, Liu SI, Stadlin A. Association analysis of GABA receptor subunit genes on 5q33 with heroin dependence in a Chinese male population. *American journal of medical genetics Part B, Neuropsychiatric genetics : the official publication of the International Society of Psychiatric Genetics* 2007; **144b**(4)**:** 439-443.

95. Vereczkei A, *et al*. Multivariate analysis of dopaminergic gene variants as risk factors of heroin dependence. *PloS one* 2013; **8**(6)**:** e66592.

96. Wang N, *et al*. Association between dopamine D2 receptor gene polymorphisms and the risk of heroin dependence. *Genetics and molecular research : GMR* 2016; **15**(4).

97. Cai M, *et al*. Association between the traditional Chinese medicine pathological factors of opioid addiction and DRD2/ANKK1 TaqIA polymorphisms. *BMC complementary and alternative medicine* 2015; **15:** 209.

98. Mehic-Basara N, Oruc L, Kapur-Pojskic L, Ramic J. Association of dopamine receptor gene polymorphism and psychological personality traits in liability for opioid addiction. *Bosnian journal of basic medical sciences / Udruzenje basicnih mediciniskih znanosti = Association of Basic Medical Sciences* 2013; **13**(3)**:** 158-162.

99. Hou QF, Li SB. Potential association of DRD2 and DAT1 genetic variation with heroin dependence. *Neuroscience letters* 2009; **464**(2)**:** 127-130.

100. Doehring A, *et al*. Genetic variants altering dopamine D2 receptor expression or function modulate the risk of opiate addiction and the dosage requirements of methadone substitution. *Pharmacogenetics and genomics* 2009; **19**(6)**:** 407-414.

101. Deng XD, *et al*. Association between DRD2/ANKK1 TaqIA polymorphism and common illicit drug dependence: evidence from a meta-analysis. *Human immunology* 2015; **76**(1)**:** 42-51.

102. Chen D, *et al*. Association between polymorphisms of DRD2 and DRD4 and opioid dependence: evidence from the current studies. *American journal of medical genetics Part B, Neuropsychiatric genetics : the official publication of the International Society of Psychiatric Genetics* 2011; **156b**(6)**:** 661-670.

103. Chien CC, Lin CH, Chang YY, Lung FW. Association of VNTR polymorphisms in the MAOA promoter and DRD4 exon 3 with heroin dependence in male Chinese addicts. *The world journal of biological psychiatry : the official journal of the World Federation of Societies of Biological Psychiatry* 2010; **11**(2 Pt 2)**:** 409-416.

104. Kotler M, *et al*. Excess dopamine D4 receptor (D4DR) exon III seven repeat allele in opioid-dependent subjects. *Molecular psychiatry* 1997; **2**(3)**:** 251-254.

105. Li T, *et al*. Association analysis of the dopamine D4 gene exon III VNTR and heroin abuse in Chinese subjects. *Molecular psychiatry* 1997; **2**(5)**:** 413-416.

106. Oosterhuis BE, *et al*. Catechol-O-methyltransferase (COMT) gene variants: possible association of the Val158Met variant with opiate addiction in Hispanic women. *American journal of medical genetics Part B, Neuropsychiatric genetics : the official publication of the International Society of Psychiatric Genetics* 2008; **147b**(6)**:** 793-798.

107. Horowitz R, *et al*. Confirmation of an excess of the high enzyme activity COMT val allele in heroin addicts in a family-based haplotype relative risk study. *American journal of medical genetics* 2000; **96**(5)**:** 599-603.

108. Cheng CY, *et al*. Brain-derived neurotrophic factor (Val66Met) genetic polymorphism is associated with substance abuse in males. *Brain research Molecular brain research* 2005; **140**(1-2)**:** 86-90.

109. Hou H, *et al*. Influence of brain-derived neurotrophic factor (val66met) genetic polymorphism on the ages of onset for heroin abuse in males. *Brain research* 2010; **1353:** 245-248.

110. Jia W, *et al*. Polymorphisms of brain-derived neurotrophic factor associated with heroin dependence. *Neuroscience letters* 2011; **495**(3)**:** 221-224.

111. Jin T, *et al*. The relationship between polymorphisms of BDNFOS and BDNF genes and Heroin addiction in the Han Chinese population. *The journal of gene medicine* 2016.

112. Haerian BS. BDNF rs6265 polymorphism and drug addiction: a systematic review and meta-analysis. *Pharmacogenomics* 2013; **14**(16)**:** 2055-2065.

113. Levran O, *et al*. Nerve growth factor beta polypeptide (NGFB) genetic variability: association with the methadone dose required for effective maintenance treatment. *The pharmacogenomics journal* 2012; **12**(4)**:** 319-327.

114. Wang Y, *et al*. A population-based association study of casein kinase 1 epsilon loci with heroin dependence in Han Chinese. *Journal of molecular neuroscience : MN* 2014; **53**(2)**:** 143-149.

115. Levran O, *et al*. Stress-related genes and heroin addiction: a role for a functional FKBP5 haplotype. *Psychoneuroendocrinology* 2014; **45:** 67-76.

116. Sun Y, *et al*. MAOA rs1137070 and heroin addiction interactively alter gray matter volume of the salience network. *Scientific reports* 2017; **7:** 45321.

117. Sun Y, *et al*. ZNF804A variants confer risk for heroin addiction and affect decision making and gray matter volume in heroin abusers. *Addiction biology* 2016; **21**(3)**:** 657-666.

118. Hancock DB, *et al*. Replication of ZNF804A gene variant associations with risk of heroin addiction. *Genes, brain, and behavior* 2015; **14**(8)**:** 635-640.

119. Human genomics. The Genotype-Tissue Expression (GTEx) pilot analysis: multitissue gene regulation in humans. *Science (New York, NY)* 2015; **348**(6235)**:** 648-660.

120. Kundaje A, *et al*. Integrative analysis of 111 reference human epigenomes. *Nature* 2015; **518**(7539)**:** 317-330.
